# Chemical Constituents and Antidepressant-Like Effects in Ovariectomized Mice of the Ethanol Extract of *Alternanthera philoxeroides*

**DOI:** 10.3390/molecules23092202

**Published:** 2018-08-31

**Authors:** Charinya Khamphukdee, Orawan Monthakantirat, Yaowared Chulikhit, Suradet Buttachon, Michael Lee, Artur M. S. Silva, Nazim Sekeroglu, Anake Kijjoa

**Affiliations:** 1 Graduate School of Pharmaceutical Sciences, Khon Kaen University, Khon Kaen 40002, Thailand; jarin_jd@yahoo.com; 2 Division of Pharmaceutical Chemistry, Faculty of Pharmaceutical Sciences, Khon Kaen University, Khon Kaen 40002, Thailand; yaosum@kku.ac.th; 3 ICBAS-Instituto de Ciências Biomédicas Abel Salazar, Rua de Jorge Viterbo Ferreira, 228, 4050-313 Porto, Portugal; nokrari_209@hotmail.com; 4 Interdisciplinary Centre of Marine and Environmental Research (CIIMAR), Terminal de Cruzeiros do Porto de Lexões, Av. General Norton de Matos s/n, 4450-208 Matosinhos, Portugal; 5 Department of Chemistry, University of Leicester, University Road, Leicester LE 7 RH, UK; ml34@leicester.ac.uk; 6 Departamento de Química & QOPNA, Universidade de Aveiro, 3810-193 Aveiro, Portugal; artur.silva@ua.pt; 7 Department of Food Engineering, Faculty of Architecture and Engineering, Kilis 7 Aralık University, Kilis 79000, Turkey; nsekeroglu@gmail.com

**Keywords:** *Althernanthera philoxeroides*, estrogenic activity, antidepressant-like effects, MAO inhibitor, ovariectomized mice, *C*-glycosylated flavones, *C*-*E*-propenoic substituted flavones

## Abstract

The previously unreported flavone glycoside, demethyltorosaflavone B (**2**) and the *E*-propenoic acid substituted flavone, torosaflavone E (**3a**), were isolated together with nine previously reported metabolites, including indole-3-carbaldehyde, oleanonic acid, vanillic acid, *p*-hydroxybenzoic acid, altheranthin (**1a**), alternanthin B (**1b**), demethyltorosaflavone D (**3b**), luteolin 8-*C*-*E*-propenoic acid (**4**) and chrysoeriol 7-*O*-rhamnoside (**5**), from the ethanol extract of the aerial part of *Althernanthera philoxeroides*. The crude ethanol extract was evaluated for its in vitro estrogenic activity in MCF-7 breast cancer cell line. The crude ethanol extract was also investigated *in vivo* for its antidepressant-like effects on ovariectomized mice using tail suspension and forced swimming tests, while its effect on the locomotor activity was evaluated by a Y-maze test. The effect of the crude extract on the serum corticosterone level, size and volume of uterus of the ovariectomized mice were also investigated. The expression of the mouse cyclic adenosine monophosphate (cAMP) response element-binding protein (CREB), brain-derived neurotrophic factor (BDNF) and β-actin mRNAs in hippocampus and frontal cortex was also evaluated, using semiquantitative reverse transcription-polymerase chain reaction. The crude extract and the isolated compounds **1a**, **1b**, **3a**, **3b** and **5,** were evaluated for their inhibitory effects on monoamine oxidases (MAOs)-A and -B.

## 1. Introduction

**Figure 1:**
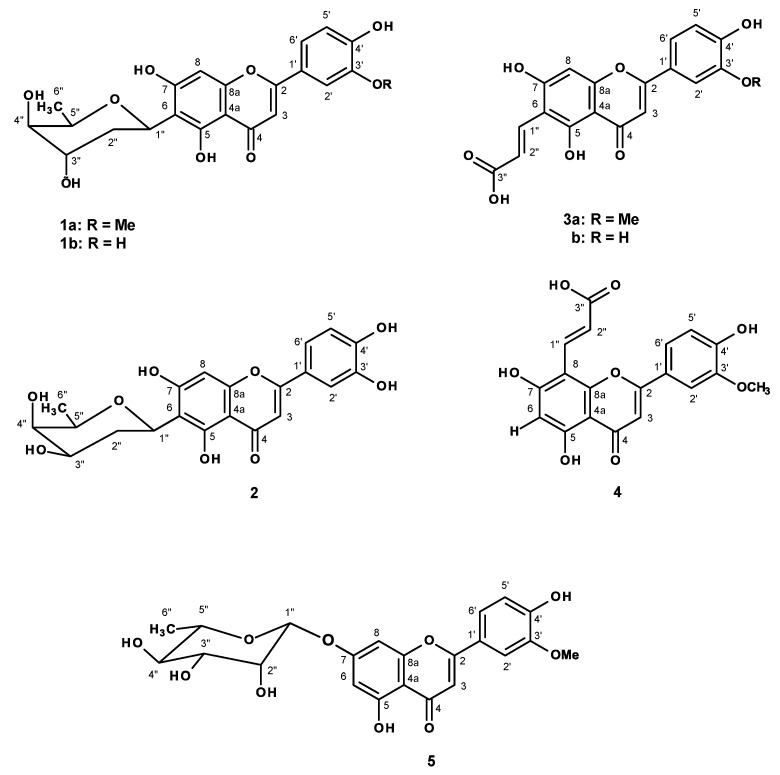
Flavonoid constituents of *Alternanthera philoxeroides*.

*Althernanthera philoxeroides* (Mart.) Griseb (Family Amaranthaceae), commonly known as alligator weed, is used in Chinese folk medicine for the treatment of acute brain fever, measles, and herpes zoster [1]. The decoction of this plant is also used for treatment of anemia in India [2]. This plant is also used for hazy vision, night blindness, malaria, post-natal complaints, diarrhea, dysentery and puerperal fever in Rajshahi, Bangladesh [3]. The ethanol extract of leaf of *A. philoxeroides* was found to exhibit anti-inflammatory and anti-arthritic activities by stabilizing the human red blood cell membrane [4]. Previous chemical studies of *A. philoxeroides* described the isolation of several bioactive compounds such as cytotoxic pentacylic triterpene saponins philoxeroidesides A–D [1], antiviral pentacyclic triterpenes chikusetsusaponin IVa and calenduloside E [5], and *C*-glycosylated flavone alternanthin [6] and alternanthin B [1], as well as phenolics, including kaempferol, ferulic acid, salicylic acid, syringic acid and chlorogenic acid [7]. In Thai folk medicine, *A. philoxeroides* and *A. sessilis* are collectively known as “Phak Pet” and are used indiscriminately as an ingredient of a decoction to ameliorate blood conditions and to stimulate milk secretion, as well as to treat post-natal depression. However, there are no pharmacological studies to support these medicinal claims so far. Therefore, the effect of the crude ethanol extract of *A. philoxeroides* was investigated for its capacity to attenuate the depression-like behaviors in the ovariectomized (OVX) mice model using the tail suspension (TST) and forced swimming tests (FST). Additionally, the estrogenic activity of the *A. philoxeroides* crude extract was evaluated through the proliferation of the MCF-7 breast cancer cell line. The molecular mechanism underlying this pharmacological properties of the extract was investigated by determination of the expression of mouse cyclic AMP response element-binding protein (CREB), brain-derived neurotrophic factor (BDNF) and β-actin m RNAs in hippocampus and frontal cortex using reverse transcription-polymerase chain reaction. Moreover, the crude ethanol extract of *A. philoxeroides* and some of its flavonoid constituents (Figure 1) were evaluated for their capacity to inhibit monoamine oxidases (MAOs)-A and -B to support the antidepressant activity of this plant in OVX mice.

## 2. Results

### 2.1. Chemical Investigation of the Crude Ethanol Extract of A. philoxeroides

Partition of the crude ethanol extract of *A. philoxeroides* with CHCl_3_ and EtOAc, followed by fractionation by column chromatography and further purification of the CHCl_3_ extract resulted in the isolation of indole-3-carbaldehyde [8] and oleanonic acid [9], while the EtOAc extract furnished vanillic acid, *p*-hydroxybenzoic acid (Supplementary Materials, Figure S1), and the previously reported *C*-glycosyl flavonoids altheranthin (**1a**) [6] and alternanthin B (**1b**) acid [1], a propenoic acid substituted flavone demethyltorosaflavone D (**3b**) [10] and luteolin 8-*C*-*E*-propenoic acid (**4**) [11], and chrysoeriol 7-*O*-rhamnoside (**5**) [12], in addition to two previously undescribed demethyltorosaflavone B (**2**), and torosaflavone E (**3a**) (Figure 1). 

### 2.2. Estrogenic Activity of the Ethanol Crude Extract of A. Shiloxeroides in MCF-7 Cell

The ethanol crude extract of *A. philoxeroides* was assayed for its estrogenic activity on MCF-cell proliferation with the concentrations ranging from 1 to 100 μg/mL. EqE_1_, EqE_10_ and EqE_100_ (the concentrations that stimulated cell proliferation equivalent to 1, 10 and 100 pM of 17β-estradiol, respectively) were determined according to Monthakanthirat et al. [13]. The crude ethanol extract at the concentration 1.68 μg/mL was found to be as effective as 100 pM of 17β-estradiol (EqE_100_ = 1.68 μg/mL).

### 2.3. The Effect of the Ethanol Crude Extract of A. philoxeroides on Ovariectomized-Induced Depressive-like Behaviors

**Figure 2:**
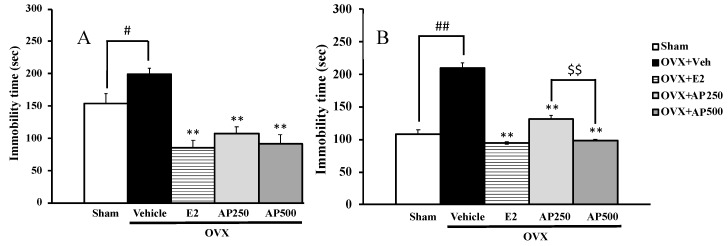
The effect of a daily administration of 17β-estradiol (E2) and a crude ethanol extract of *A. philoxeroides* on ovariectomized (OVX)-induced hopeless behaviors in forced swimming tests (FST) (**A**) and tail suspension test (TST) (**B**). Immobility time of each animal group was measured as an index of depression. Each data column represents the mean ± SEM (*n* = 8–10 in each animal group). ^#^ *p* < 0.05 and ^##^ *p* < 0.01 compared with the vehicle-treated sham group (*t*-test), ** *p* < 0.01 compared with the vehicle-treated OVX group (Tukey test) and ^$$^ *p* < 0.01 compared between different doses.

The depressive-like symptoms of the OVX mice were evaluated using FST and TST (Figure 2A,B). The vehicle-treated OVX group exhibited significantly longer immobility time than the sham group. On the contrary, the 17β-estradiol (E2) and ethanol crude extract of *A. philoxeroides*-treated OVX groups showed a significantly shorter immobility time than the vehicle-treated OVX group. 

**Figure 3:**
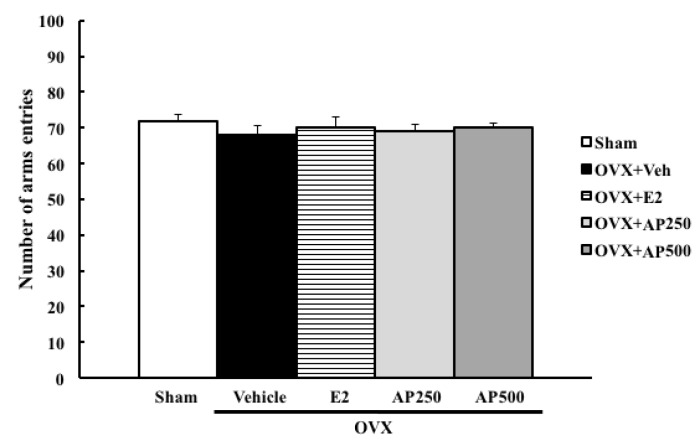
The effect of a daily administration of 17β-estradiol (E2) and ethanol crude extract of *A. philoxeroides* on a locomotor activity in a Y-maze test. The number of arms entries of each animal group was determined. Each data column represents the mean ± SEM (*n*= 8–10 in each animal group).

Moreover, the locomotor activity, using a Y-maze task, was also performed to exclude false positive results. The results showed that neither 17β-estradiol (E2) nor the ethanol crude extract of *A. philoxeroides* altered the locomotive activity of OVX mice (Figure 3).

### 2.4. 17β-Estradiol Levels and the Effect of the Ethanol Crude Extract of A. phliroxeroides on Uterine Weight and Volume

The levels of serum 17β-estradiol were evaluated (after decapitation) by electrochemiluminescence immunoassay. The level of 17β-estradiol in the vehicle-treated OVX group was significantly different in all of the treatment-groups, confirming the efficacy of the surgical procedure of ovariectomy, which involved estrogen deprivation. The weight and volume of the uterus were determined as an index of estrogen receptors activation. Results shown in Table 1 revealed a statistically significant effect of estrogen deprivation in OVX mice (*p* < 0.001). For the treatment groups, both the ethanol crude extract of *A. philoxeroides* (250 and 500 mg/kg/day) and 17β-estradiol (E_2_) (1 µg/kg/day) showed statistically significant effects on uterus weight and volume.

### 2.5. Effects of 17β-Estradiol and the Ethanol Crude Extract of A. philoxeroides on HPA-Axis via Serum Corticosterone Level

**Figure 4:**
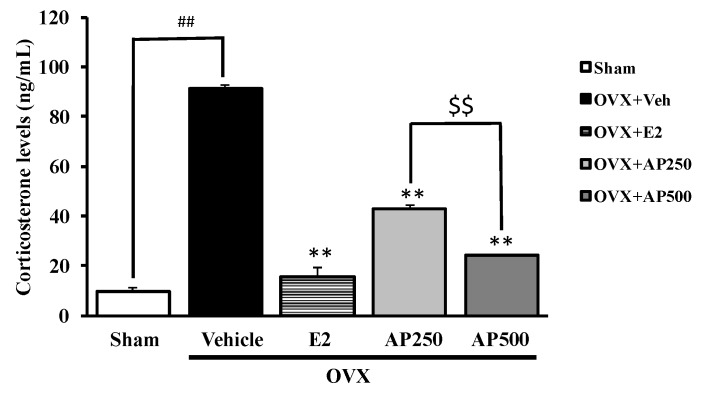
The effect of the ethanol crude extract of *A. philoxeroides* on the corticosterone level. Each column represents the mean ± SEM (*n* = 3–5). ^##^ *p* < 0.001 vs. sham-operated group, ** p < 0.001 vs. OVX group and ^$$^ *p* < 0.001 vs. *A. philoxeroides*-treated group (post hoc Tukey test).

The serum corticosterone was evaluated to clarify the feedback mechanism in the HPA-axis on the estrogen deprivation in the OVX mice. The results showed significant effect of estrogen deprivation in OVX mice on HPA-axis by an increase of serum corticosterone level (*p* < 0.001). For the treatment group, both the ethanol crude extract of *A. philoxeroides* (250 and 500 mg/kg/day) and 17β-estradiol (E2) (1 µg/kg/day) showed a significant effect on HPA-axis by decreasing the serum corticosterone level (*p* < 0.001) when compared to the vehicle-treated OVX group. Moreover, the ethanol crude extract of *A. philoxeroides* (250 and 500 mg/kg/day) significantly decreased serum corticosterone level (*p* < 0.001) in a dose-dependent manner, as showed in Figure 4.

### 2.6. The Ethanol Crude Extract of A. philoxeroides Ameliorates OVX-Induced Dysfunction of BDNF-CREB Systems

**Figure 5:**
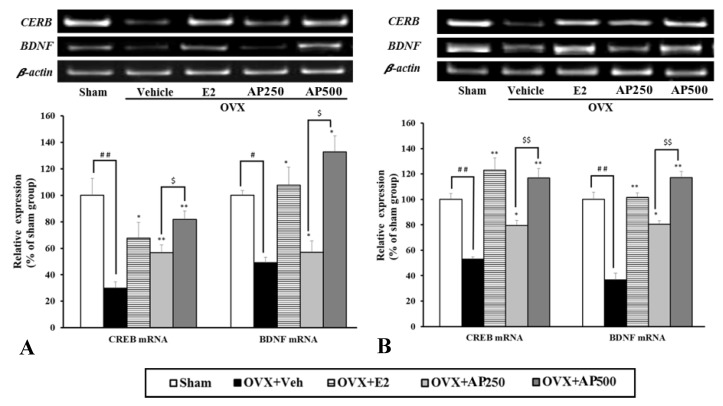
Effect of the *A. philoxeroides* extract on the expression levels of cyclic AMP response element-binding protein (CREB) and brain-derived neurotrophic factor (BDNF) mRNAs in the hippocampus (**A**) and frontal cortex (**B**) of sham and OVX mice. The expression of BDNF and CREB mRNAs were semi-quantitatively analyzed as described in the text. Each data column represents the mean ± SEM (*n* = 4–6 in each animal group). # *p* < 0.05, ^##^ *p* < 0.01, compared with the vehicle-treated sham group (*t*-test), * *p* < 0.05, ** *p* < 0.01, compared with vehicle-treated OVX group (Tukey test) and ^$^ *p* < 0.05 and ^$$^ *p* < 0.01 compared between different doses.

Semi-quantitative analysis of CREB and BDNF mRNA expression revealed that, in the OVX mice, the expression levels of these genes in the hippocampus and frontal cortex were significantly down-regulated, as compared with those in the sham operation group; however, they were reversible by 17β-estradiol treatment (Figure 5).

As with the estrogen treatment, the administration of *A. philoxeroides* extract (250 and 500 mg/kg/day) dose-dependently normalized the down-regulated expression of these genes in the OVX mice.

### 2.7. MAO-A and MAO-B Inhibitory Activity of the Ethanol Crude Extract of A. philoxeroides and Some of Its Flavonoid Constituents

The crude ethanol extract of *A. philoxeroides,* together with its constituents, alternanthin (**1a**), althernanthin B (**1b**), torosaflavone E (**3a**), dimethyltorosaflavone D (**3b**) and chrysoeriol 7-rhamnoside (**5**) were tested for their inhibitory activity on MAO-A and MAO-B, using the recombinant human MAO-A and MAO-B. The rates of MAO catalysis was determined by measuring the production of 4-hydroxyquinoline by fluorescence spectrophotometry. Both ethanol crude extract of *A. philoxeroides* and the tested compounds showed inhibitory effects against MAO-A and MAO-B. The IC_50_ values were converted to the corresponding enzyme-inhibitor dissociation constants (*Ki* values) using the Cheng–Prusoff equation [14]; *Ki* = IC_50_/(1 + [S]/*Km*). For this purpose, *Km* values for the oxidation of kynuramine by MAO-A and MAO-B were used from previous studies, i.e., 16.1 μM for human MAO-A and 22.7 μM for human MAO-B [15]. The *Ki* values enabled the calculation of the MAO-A/B selectivity ratios (Si = *Ki* (MAO-B/*Ki* (MAO-A)). The results are showed in Table 2. The selectivity index for the MAO-A and MAO-B isoform indicates that *A. philoxeroides* extract, althernanthin B (**1b**), torosaflavone E (**3a**), dimethyltorosaflavone D (**3b**), chrysoeriol 7-rhamnoside (**5**) are partially selective for MAO-B isoform whereas alternanthin (**1a**) is partially selective for MAO-A isoform.

## 3. Discussion

It is well recognized that estrogen has an important role in the control of mammalian female’s mood and behavior. Clinical and experimental evidences have suggested that alteration of sexual hormones may contribute to depressive disorders which are more than twice as prevalent in women as in men [16]. Moreover, fluctuations in ovarian hormones after a significant decline in estrogen level in situations such as menopausal transition, post-menopausal status and/post-ovariectomy, may be associated with episodes of reduced energy, lowered mood and decrease of activity. Estrogen can be used as an adjunct therapy for attenuation of the depressive symptoms during the perimenopause and postpartum in patients who do not respond to conventional antidepressant treatment [17]. Estrogen replacement therapy (ERT) also improves mood alteration and reduces anxiety in some antidepressant-naive perimenopausal women with clinical depression [18]. Moreover, preclinical studies revealed that estrogen can exert anxiolytic and antidepressant-like effects in bilaterally OVX mice in depression-despair animal tests such as FST and TST [19]. 17β-Estradiol can also reduce the latency to the onset of action for some antidepressants in the FST [20]. Although the mechanism for the effect of 17β-estradiol is still not well understood, it was suggested that this compound exerted an antidepressant-like effect, preferentially through the modulation of dopaminergic and serotonergic receptors [21]. Moreover, ERT was found to modulate the function of HPA-axis [22], which exerts antidepressant and anxiolytic effects in women. Another antidepressant mechanism of estrogen could be related to its neurotrophic and neuroprotective effects in the hippocampus; in particular, it has been documented that the administration of 17β-estradiol in OVX mice induces an increase in the release of BDNF [23] and vascular endothelial growth factor (VEGF) [24], thus decreasing depressive-like behaviors [25]. However, a potential damaging health risk profile including an increased risk of breast or endometrial cancer and thromboembolic events, associated with a long-term treatment with estrogen, has limited its use in high-risk populations. Due to a negative effect of ERT, many researchers have looked for naturally occurring compounds that can minimize postmenopausal syndrome [26,27]. In this context, many flavonoid-containing plants seem to be good candidates, as a number of flavonoids have proven to have estrogenic activity [28].

Since *Althernanthera philoxeroides* is used in Thai folk medicine to ameliorate blood conditions and to treat post-natal depression, we have investigated the effect of its ethanol crude extract in the OVX mice model. Since the bilateral ovariectomy of mice induced depression-like behaviors in both FST and TST, the crude ethanol extract of *A. philoxeroides* was tested for its effect on OVX-induced depressive disorder by both tests. It is well known that the estrogen depletion caused by ovariectomy contributes to an impairment of mood function, thus an increase in immobility time in both tests can be used as an index of hopelessness or despair, which is also a major symptom exhibited by patients with depression and menopause. The duration of immobility is evaluated in both tests to assess the depressive behaviors and selective response to depression in dopaminergic and serotonergic receptors [29]. The results revealed that treatment of the OVX mice with 17β-estradiol (1 μg/kg/day) and the crude ethanol extract of *A. philoxeroides* (250 and 500 mg/kg/day) for eight weeks significantly decrease the immobility times in both FST and TST without any effect on locomotor activity (Figure 2) when compared to the vehicle-treated group. Moreover, it was found that early and repeated administration of the extract of *A. philoxeroides* and 17β-estradiol ameliorated OVX-induced depressive behaviors. The effect of *A. philoxeroides* extract can be attributed to its chemical constituents, consisting mainly in flavone derivatives which can act as phytoestrogens [30]. Therefore, these findings can serve as a scientific-based evidence for the claim in Thai folk medicine of the property of *A. philoxeroides* for the treatment of mood disorder in menopause and postpartum. 

In order to ensure that flavonoids could be responsible for this activity, we have investigated the chemical constituents of the crude ethanol extract of *A. philoxeroides.* Chromatographic fractionation and further purification procedures resulted in the isolation of five previously described flavone derivatives, i.e., althernanthin (**1a**) [6], alternanthin B (**1b**) acid [1], demethyltorosaflavone D (**3b**) [10], luteolin 8-*C*-*E*-propenoic acid (**4**) [11], and two previously unreported demethyltorosaflavone B (**2**), and torosaflavone E (**3a**) (Figure 1), in addition to the known indole-3-carbaldehyde [8], oleanonic acid [9], vanillic acid, *p*-hydroxybenzoic acid. The structures of all the known compounds were elucidated by analysis of their 1D and 2D-NMR spectra as well as HRMS data, and also by comparison of their spectral data to those reported in the literature. In the case of althernanthin (**1a**), the X-ray analysis was also performed to confirm the structure and the absolute configurations of all the stereogenic centers of the sugar moiety (Supplementary Materials, Figure S30).

Compound **2** was isolated as a yellow amorphous solid (mp 232–233 °C), and its molecular formula C_21_H_20_O_9_ was established based on its (+)-HRESIMS *m*/*z* 417.1210 [M + H]^+^, (calculated 417.1186 for C_21_H_21_O_9_), indicating twelve degrees of unsaturation. The ^13^C-NMR spectrum of **2** displayed 21 carbon signals which, according to DEPTs and HSQC spectra (Table 3, Supplementary Materials, Figures S15 and S17), can be classified as one conjugated ketone carbonyl (δ_C_ 181.9), six oxyquaternary sp^2^ (δ_C_ 164.1, 162.0, 157.5, 156.1, 150.0, 145.8), three quaternary sp^2^ (δ_C_ 121.5, 110.1, 103.0), four methine sp^2^ (δ_C_ 119.1, 116.0, 113.4, 94.6), four oxymethine sp^3^ (δ_C_ 74.4, 70.1, 69.6, 68.5), one methylene sp^3^ (δ_C_ 32.3) and one secondary methyl (δ_C_ 17.4) carbons. The ^1^H-NMR spectrum (Table 3, Supplementary Materials, Figure S14) exhibited a double doublet at δ_H_ 7.43 (*J* = 8.4, 2.2 Hz, H-6′), two doublets at δ_H_ 6.89 (*J* = 8.4 Hz, H-5′) and 7.41 (*J* = 2.2 Hz, H-2′), two singlets at δ_H_ 6.70 (H-3) and 6.52 (H-8), characteristic of a 3-substituted 5, 7, 3′, 4′-tetrahydroxy flavone, in addition to a double doublet at δ_H_ 4.99 (*J* = 11.9, 2.9 Hz, H-1′′), a quartet at δ_H_ 3.65 (*J* = 6.4 Hz, H-5′′), a multiplet at δ_H_ 3.71 (H-3′′), two multiplets of two methylene protons at δ_H_ 2.05 and 1.60 (H_2_-2′′) and a doublet of the methyl protons at δ_H_ 1.18 (*J* = 6.4 Hz, H_3_-6′′), in addition to a singlet of a hydrogen-bonded hydroxyl group at δ_H_ 13.53 (OH-5). The ^1^H- and ^13^ C-NMR data of **2** are very similar to those of torosaflavone B, a *C*-2,6-dideoxyglycosylflavone, isolated from leaves of *Cassia torosa* Cav. [31], except for the absence of a methoxyl group. The COSY and HMBC spectra (Table 3, Supplementary Materials, Figures S16 and S18) confirmed that the flavone moiety was 5,6,3′,4′-tetrahydroxyflavone. That C-6 of the flavone moiety was α-glycosylated was supported by HMBC correlations from the anomeric α-proton (H-1′′) to C-6 (δ_C_ 110.1). The sugar moiety was identified as 2, 6-dideoxylyxohexose (oliose) by comparison of its ^1^H and ^13^C chemical shifts with those of the sugar moiety of torosaflavone B [31] as well as by COSY and HMBC correlations (Table 3). Therefore, **2** was identified as demethyltorosaflavone B. The literature search revealed that **2** has never been previously reported.

Compound **3a** was also isolated as a yellow crystal (mp 250-252 °C) and its molecular formula C_19_H_14_O_8_ was established on the basis of its (+) ESI *m*/*z* 371 (M + H)^+^ and (+)-HRESIMS *m*/*z* 353.0658 (M − H_2_O + H)^+^ (calculated 353.0661 for C_19_H_13_O_7_), indicating thirteen degrees of unsaturation. The ^13^C-NMR spectrum (Table 4, Supplementary Materials, Figure S20) displayed nineteen carbon signals which, in combination with DEPTs and HSQC spectra (Supplementary Materials, Figure S22), can be categorized as one conjugated ketone carbonyl (δ_C_ 181.4), one conjugated carboxyl (δ_C_ 168.8), six oxyquaternary sp^2^ (δ_C_ 163.7, 163.5, 161.8, 157, 4, 150.9, 148.1), three quaternary sp^2^ (δ_C_ 121.3, 106.0, 102.8), seven methine sp^2^ (δ_C_ 134. 0, 120.5, 119.6, 115.8, 110.2, 103.1, 94.0) and one methoxyl (δ_C_ 56.0) carbons. The ^1^H-NMR spectrum (Table 4, Supplementary Materials, Figure S19) exhibited, in addition to the proton signals of the 5,7,3′,4′-tetraoxygenated-6-alkyl flavone at δ_H_ 7.58, dd (*J* = 8.2, 2.1 Hz, H-6′; δ_C_ 120.5), 7.57, brs (H-2′; δ_C_ 110.2), 6.96, s (H-3; δ_C_ 103.6), 6.95, d (*J* =8.2 Hz, H-5′; δ_C_ 115.8), 6.55, brs (H-8; δ_C_ 94.0), two doublets of a *trans* double bond at δ_H_ 7.91, d (*J* = 16.2 Hz, H-1′′; δ_C_ 134.0)/ δ_H_ 6.86, d (*J* = 16.2 Hz, H-2′′; δ_C_ 119.6) and a singlet of a hydroxyl group at δ_H_ 14.45 of the *E*-propenoic acid moiety. That the *E*-propenoic moiety was on C-6 of the flavone nucleus was substantiated by HMBC correlations (Table 4, Supplementary Materials, Figure S23) from H-1′′ to C-5 and C-7. Literature search revealed that the NMR data of **3a** resembled to those of torosaflavone D, previously isolated from the leaves of *Cassia torosa* Cav. [32]. However, the positions of the OH and OCH_3_ groups of the flavone nucleus were interchanged. In torosaflavone D, the methoxyl group is on C-4′ and the OH group is on C-3′. However, in **3a** the OCH_3_ is on C-3′ and OH is on C-4′. This was supported by a strong HMBC correlations from H-5′ to the methoxyl bearing carbon C-3′ (δ_C_ 148.1) and weak HMBC correlation to C-4′ (δ_C_ 150.9). This substitution pattern was confirmed by the NOESY correlation from OMe-3′ (δ_H_ 3.89, s) to H-2′ and not to H-5′. Therefore **3a** is a new compound which was named torosaflavone E.

In order to clarify the mechanism by which a treatment with *A. philoxeroides* extract relieved the depressive symptom in OVX mice, the weight and volume of the uterus of the *A. philoxeroides* extract-treated OVX mice were compared with those of the vehicle-treated OVX mice. The hypertrophy of the uterus in OVX mice was a classical end point used as an indirect measurement of the estrogenic potency of the endocrine disrupting chemicals in the OVX rodent model. The results showed that both 17β-estradiol and the *A. philoxeroides* extract exerted the estrogenic-like activity by increasing the weight and volume of the uterus when compared to the vehicle-treated OVX mice (Table 1). These results agree with those observe in the in vitro study showing that the *A. philoxeroides* extract promotes cell proliferation of the MCF-7 breast cancer cell line. Moreover, it was found that the uterotrophic effects of 17β-estradiol were, in part, due to an activation of the expression of three estrogen responsive genes in uterus, i.e. insulin-like growth factor 1 (IGF-1), progesterone receptor (PR), and complement protein 3 (C3). In turn, the activation of the uterine IGF-1 expression stimulates the proliferation of uterine tissues such as myometrium and endometrium [33]. Interestingly, the flavanone 8-prenylnaringenin, in high dose, also stimulated serum prolactin levels, uterine weight, and progesterone receptor, IGF- 1 and complement protein C3 mRNA transcripts [34]. However, the effects of the *A. philoxeroides* extract should be further investigated to clarify if it also activates the uterine IGF-1 expression and estrogen receptor. In addition, the histopathological test of the uterus should be performed to assess the effect of this extract.

To provide insight into the effect of the treatment with 17β-estradiol and the *A. philoxeroides* extract in ameliorating the depressive-like symptoms in OVX mice, the serum corticosterone levels, as a marker of hyperactivation at the HPA-axis, were analyzed. The results showed that OVX mice contained significantly higher serum corticosterone level than that of the sham-operated group (Figure 4), suggesting that the HPA-axis is activated in OVX mice. However, OVX-induced changes in serum corticosterone levels remain controversial, as previous studies have demonstrated that ovariectomy exerted no effect on serum corticosterone level or even decreased it when compared to the control; however, the reason for this discrepancy is still unclear [35,36]. On the contrary, our results showed that OVX-induced elevation in serum corticosterone levels (Figure 4), which was accompanied by depression-like behaviors, was similar to stress-induced endcrine responses and behavioral changes. Previous investigations indicated that patients with depression showed hyperactivity of the HPA-axis which is attributable to an impaired feedback mechanism of the HPA-axis [37]. Although the mechanism underlying the effect of the *A. philoxeroides* extract needs to be further investigated, a speculative explanation is that it could prevent OVX-induced dysfunction in a negative feedback mechanism of the HPA-axis. This hypothesis seems to be supported by previous findings which showed an interference of 17β-estradiol with the activity of neurotransmitters regulating the secretion of corticotrophin-releasing factor (CRF) from the hypothalamus [38]. Our findings were also supported by the fact that chronic administration of flavonoids extracted from a Chinese herbal decoction ameliorated behavioral alterations and hippocampal dysfunctions as well as significantly decreased serum corticosterone level and its upstream stress hormone adrenoc orticotropic hormone (ACTH) level in chronically stressed rats [39].

Neurogenesis and neuroplasticity are found to be strongly related to pathology of degenerative diseases associated with estrogen level [40]. Therefore, the *A. philoxeroides* extract was investigated for its molecular and cellular mechanisms by estrogen receptor-mediated reinforcement of CREB and BDNF genes transcription. BDNF plays an important role in neuronal survival and growth, serves as a neurotransmitter modulator, and participates in neuronal plasticity in the hippocampus and frontal cortex, which is essential for learning and memory [41]. It binds to the high affinity receptor TrkB (tyrosine kinase B) and activates signal transduction cascades (IRS1/2, PI3K, Akt), crucial for CREB production [42]. The decrease of BDNF level will impair the function of neuron and leads to the occurrence of depression [43]. The results revealed that ovariectomy down-regulated the expression of BDNF mRNA in the hippocampus and frontal cortex which was reversed by treatment with 17β-estradiol. These findings are consistent with those reported by Monthakantirat et al. [44]. Moreover, OVX mice treated with the *A. philoxeroides* extract exhibited the up-regulation of BDNF and CREB mRNA expression in a dose-dependent manner in both hippocampus and frontal cortex. Therefore, it is likely that the *A. philoxeroides* extract ameliorated depression-like behaviors in OVX mice via stimulation of CREB- and BDNF-mediated neuroplasticity and neurogenesis in the hippocampus and frontal cortex. Interestingly, there are evidences that high-flavonoid intake induces cognitive improvements linked to changes in serum BDNF [45,46]. Therefore, it is probable that the flavonoid constituents of the ethanol extract of *A. philoxeroides* are responsible for the increased level of BDNF in the OVX mice treated with the *A. philoxeroides* extract. Furthermore, our findings also supported the hypothesis that the high corticosterone level is associated with the down-regulated transcription of CREB and BDNF genes [47].

On the other hand, monoamine oxidases (MAOs) are the key enzymes involved in catecholamine and serotonin neurotransmitters such as dopamine, norepinephrine and epinephrine which are related to depression and cognitive dysfunction [48]. The crude ethanol extract of *A. philoxeroides* was found to inhibit both MAO-A and MAO-B with IC_50_ values of 252.9 and 90.69 μg/mL, respectively. These values are comparable to those exhibited by the extract of *Hypericum perferatum*, i.e. IC_50_ = 120 μg/mL for MAO-A and 370 μg/mL for MAO-B [49]. The flavonoid constituents **1a**, **1b**, **3a**, **3b** and **5** were also evaluated for their inhibitory activity against MAO-A and MAO-B, together with clorgyline (selective MAO-A inhibitor) and deprenyl (selective MAO-B inhibitor). It was found that **3a** was the most potent inhibitor for MAO-B, followed by **1b**, **5** and **3b**. Interestingly, **1a** is the only compound that exhibits selectivity toward MAO-A (IC_50_ = 0.00046 uM) which is three folds more potent than clorgyline (IC_50_ = 0.00016 uM), while **1b** (IC_50_ = 0.00021 uM) was slightly less potent than clorgyline. It seems that the methoxyl group on C-3′ could play a crucial role in the difference in activity between **1a** and **1b**. Interestingly, all the compounds were more potent than deprenyl against MAO-B, being **1b** the most potent inhibitor, followed by **1a**. This is not surprising since many flavonoids have been proved to be MAOs inhibitors [50,51,52]. These findings also suggest that the antidepressant effect of the *A. philoxeroides* extract could be involved in the inhibition of MAOs activity.

## 4. Experimental Section

### 4.1. General Experimental Procedures

Melting points were determined on a Stuart Melting Point Apparatus SMP3 (Bibby Sterilin, Stone, Staffordshire, UK) and are uncorrected. Optical rotations were measured on an ADP410 Polarimeter (Bellingham + Stanley Ltd., Tunbridge Wells, Kent, UK). ^1^H- and ^13^C-NMR spectra were recorded at ambient temperature on a Bruker AMC instrument (Bruker Biosciences Corporation, Billerica, MA, USA), operating at 300 or 500 and 75 or 125 MHz, respectively. High resolution mass spectra were measured with a Waters Xevo QToF mass spectrometer (Waters Corporations, Milford, MA, USA) coupled to a Waters Aquity UPLC system. A Merck (Darmstadt, Germany) silica gel GF_254_ was used for preparative TLC, and a Merck Si gel 60 (0.2–0.5 mm) was used for column chromatography.

### 4.2. Plant Material

*Alternanthera philoxeroides* (Mart.) Griseb (family Amaranthaceae) was collected in Khon Kaen province, Thailand in May 2014. The plant material was identified by Prof. Suppachai Tiyaworanant (Department of Pharmacognosy and Toxicology, Faculty of Pharmaceutical Sciences of Khon Kaen University). The voucher specimen (KKPH010102986) was deposited at the herbarium of the Faculty of Pharmaceutical Sciences of Khon Kaen University, Thailand.

### 4.3. Extraction and Isolation of the Constituents

Dried and powdered whole plants (1.8 kg) was Soxhlet extracted with EtOH (3 × 10 L) at 50 °C for 1 h. The ethanol solution was evaporated under reduced pressure to give 176.8 g of crude ethanol extract. Part of the crude ethanol extract (100 g) was dissolved in warm EtOH (900 mL) to which was added 1L of H_2_O containing 3.6 g of lead acetate and 10.8 mL of glacial acetic acid. The solution was kept in the dark chamber for 48 h and filtered; the filtrate was concentrated at reduced pressure to remove EtOH and partitioned with CHCl_3_ (4 × 500 mL). The combined CHCl_3_ solutions were dried (anhydrous Na_2_SO_4_), filtered, evaporated at reduced pressure to give a viscous mass of CHCl_3_ extract (9.8 g). The aqueous solution was further extracted with EtOAc (4 × 500 mL) and the EtOAc solutions were combined, dried (anhydrous Na_2_SO_4_), filtered, and evaporated at reduced pressure to give a viscous mass of EtOAc extract (4.0 g). The CHCl_3_ extract was applied to a Silica gel column (145 g) and eluted with petrol-CHCl_3_, CHCl_3_ and Me_2_CO, wherein 250 mL fractions were collected as follows: Frs 1–131 (petrol-CHCl_3_, 1:1), 132–159 (petrol-CHCl_3_, 3:7), 160–210 (petrol-CHCl_3_, 1:4), 300–500 (CHCl_3_), 351–380 (Me_2_CO). Frs 160–210 were combined (320 mg) and applied on a Sephadex LH-20 column (5 g) and eluted with MeOH wherein six sub-fractions of 100 mL were collected. Sfrs 3–5 were combined and precipitated in a mixture of petrol and CHCl_3_ to give indole-3-carbaldehyde (4 mg). Frs 211–246 were combined (92.5 mg) and crystallized with a mixture of petrol and CHCl_3_ to give oleanonic acid (16 mg). The EtOAc extract was applied on a silica gel column (50 g) and eluted with petrol-CHCl_3_ and CHCl_3_-Me_2_CO, wherein 100 mL fractions were collected as follows. Frs 1–77 (petrol-CHCl_3_, 1:1), 78–102 (petrol-CHCl_3_, 3:7), 103–135 (petrol-CHCl_3_, 1:9), 136–750 (CHCl_3_-Me_2_CO, 9:1), 751–790 (CHCl_3_-Me_2_CO, 4:1), 791–1065 (CHCl_3_-Me_2_CO, 7:3), 1066–1155 (CHCl_3_-Me_2_CO, 3:2). Frs 141–145 were combined (11.4 mg) and crystallized in a mixture of petrol and CHCl_3_ to give vanillic acid (4.7 mg). Frs 150–152 were combined (17.1 mg) and crystallized in a mixture of petrol and CHCl_3_ to give *p*-hydroxybenzoic acid (4.8 mg). Frs 235–276 were combined (40 mg) and crystallized in a mixture of CHCl_3_ and Me_2_CO to give **1a** (12 mg). Frs 288–316 were combined (80.1 mg) and crystallized a mixture of CHCl_3_ and Me_2_CO to give **3a** (6 mg). Frs 317–361 were combined (58.5 mg), applied on a Sephadex LH-20 column (5 g) and eluted with a 1:1 mixture of CHCl_3_-MeOH, where in 15 sub-fractions of 50 mL were collected. Sfrs 5–13 were combined and evaporated to give further 4.3 mg of **3a** and **2** (2.6 mg). Frs 362–383 were combined (32.5 mg) and purified by TLC (Silica gel G_254_, CHCl_3_: MeOH: HCO_2_H, 9:1:0.1) to give **1b** (10.5 mg). Frs 384–443 were combined (109.2 mg) and applied on a Sephadex LH-20 column (5 g), and eluted with a 1:1 mixture of CHCl_3_/MeOH, wherein 20 sub-fractions of 50 mL fractions were collected. Sfrs 8–16 were combined and evaporated to give **1b** (12.3 mg). Frs 444–465 were combined (42.6 mg) and crystallized in a mixture of CHCl_3_ and Me_2_CO, to give **5** (6.7 mg). The mother liquor of the combined frs 444–465 was combined with frs 466–798 (260.0 mg) and applied on a Sephadex LH-20 column (5 g), and eluted with MeOH, wherein 40 sub-fractions were collected. Sfr 30–35 were combined and precipitated in MeOH to give **3b** (5 mg). The mother liquor was dried and precipitated in Me2CO to give **4** (5 mg).

#### 4.3.1. Demethyltorosaflavone B (**2**)

Yellow amorphous solid. Mp 232–233 °C (CHCl_3_/MeOH) ${\left[\alpha \right]}_{D}^{23}$-77 (MeOH, *c* 0.03 g/mL); For ^1^H and ^13^C spectroscopic data, see Table 3; HRESIMS *m*/*z* 417.1210 (M + H)^+^, cald for C_21_H_21_O_9_, 417.1186.

#### 4.3.2. Torosaflavone E (**3a**)

Yellow crystal. Mp 250–252 °C; For ^1^H and ^13^C spectroscopic data, see Table 3; ESI MH^+^ 371Da (C_19_H_15_O_8_), HRESIMS *m*/*z* 353.0658, calcd for C_19_H_13_O_7_, 353.0661(M − H_2_O + H)^+^.

### 4.4. Cell-Based Assay

The estrogenic activity was evaluated by monitoring the proliferation of cells and stimulatory potency of the extract in human breast cancer cell line (MCF-7), using the procedure described by Monthakantirat et al. [13]. MCF-7 cell line was purchased from the American Type Culture Collection (Manassas, VA, USA). The MCF-7 cells were grown in Minimum Essential Medium (MEM), supplemented with 6 ng/mL insulin, 1 mM sodium pyruvate, 1 mM nonessential amino acids, 2 mM glutamine, 10% FBS, and antibiotics (100 U/mL penicillin, 100 µg/mL streptomycin) under a 5% CO_2_ humidified atmosphere at 37 °C for three days after which the cells were transferred to the RPMI-1640 supplemented with 1 mM sodium pyruvate, 1 mM nonessential amino acids, 2 mM glutamine, 10% FBS, and antibiotics (100 U/mL penicillin, 100 µg/mL streptomycin) under a 5% CO_2_ humidified atmosphere at 37 °C. Cells were seeded into 96-well tissue culture plates in 5% dextran-coated charcoal (DCC) treated-FBS supplemented RPMI phenol red-free medium at a density of 1 × 10^4^ cells/well. The crude extract was added in 70% EtOH solution (the control contained 0.7% ethanol) and incubated at 37 °C with 5% CO_2_ for 96 h. In all experiments, serial dilutions of 17β-estradiol (Sigma-Aldrich, St. Louis, Mo, USA) were added as positive control. To evaluate relative cell concentrations, Alamar Blue reagent (Bio-Rad Laboratories Inc., Oxford, UK) was used. After 3 h, fluorescence was measured at 590 nm with excitation at 530 nm using a FL500 spectrophotometer (BIO-TEK Instruments Inc., Winooski, VT, USA). EqE_10_ and EqE_100_ represent the concentrations of the extract that stimulated the cell proliferation equivalent to 10 and 100 pM 17β-estradiol, respectively. These values were determined by linear regression analysis, which 3–5 different concentrations in quadruplicate versus ratio of cell proliferation to control were plotted.

### 4.5. Animals

Fifty female ICR mice (20–30 g, 5 weeks old) were obtained from the National Laboratory Animal Center (Mahidol University, Nakhon Pathom, Thailand). The animals were in a light-controlled room with a 12 h dark/light cycle, under controlled temperature 22 °C ± 2 °C, humidity 45% ± 2%, and were allowed free access to food and water in the Laboratory Animal Unit of the Faculty of Pharmaceutical Sciences, Khon Kaen University, Thailand. The experimental procedures used in this study were in accordance with the Guiding Principles for the Care and Use of Animals (NIH Publications No. 80–23, revised in 1996) and was also approved by the Animal Ethics Committee for Use and Care of Khon Kaen University, Khon Kaen, Thailand (Approval No. IACUC-KKU-103/60). 

### 4.6. Surgical Procedures

OVX mice were used to mimic the estrogen deprivation in animals. Ovariectomy was performed as previously described by Monthakantirat and coworkers [44]. Female mice underwent bilateral ovariectomy via dorsolateral incision under pentobarbital (60 mg/kg body weight, i.p.) anesthesia. The exposed ovary and associated oviduct were removed and then the skin incisions were closed. The sham-operated group underwent the same procedure without ovariectomy. 

### 4.7. Experimental Design

After a 3-day recovery period, the animals were divided into five groups: (1) sham, (2) ovariectomy (OVX), (3) ovariectomy + 1 μg/kg 17β-estradiol, (OVX + E2), (4) ovariectomy + 250 mg/kg of *A. philoxeroides* extract (OVX + AP250), and (5) ovariectomy + 500 mg/kg of *A. philoxeroides* extract (OVX + AP500). The sham and OVX control groups were orally administered distilled water 0.2 mL per mouse once daily for 8 weeks. 17β-Estradiol and *A. philoxeroides* extract were suspended in distilled water and orally administered 0.2 mL per mouse once daily for 8 weeks. To assess the effects of 17β-estradiol and *A. philoxeroides* extract in OVX mice, the drugs were administered 1 h before the behavioral tests. At the end of behavioral studies, the mice were anesthetized with pentobarbital sodium (Nembutal^®^) 60 mg/kg (i.p.). Thereafter, blood sera (1 mL) were collected by cardiac puncture and centrifuged (3000 rpm; 4 °C) for 15 min [53]. 17β-Estradiol and corticosterone levels were evaluated in the serum. Uteri were removed, trimmed of connective tissue and fat for measuring and weighing. Hippocampus and frontal cortex were collected for semi-quantitative reverse transcription-polymerase chain reaction (RT-PCR) assay. Both serum and all tissues were collected rapidly and kept at −80 °C for all experiments.

### 4.8. Behavioral Analysis

#### 4.8.1. Forced Swimming Test

The procedure was adapted from Vogel et al. [54]. Each mouse was placed in an individual glass cylinder (20 cm diameter, 30 cm height) containing clean water (25 ± 1 °C) to the level of 15 cm above the bottom in a quiet experimental room. On day one, mice were forced to swim in a swimming-stress session (pre-test session) for 15 min with no observation, then mice were dried with the towels. Twenty four hours after pre-test session, mice were exposed to the same experimental condition for 5 min (test session). Mice were considered immobile when remained floating and made no attempt to escape. The immobility time represented the hopeless behavior. The water was changed for each animal. During a 6 min period, the total immobility time was recorded for the last 4 min.

#### 4.8.2. Tail Suspension Test

The tail suspension test was used to assess the antidepressant effects of *A. philoxeroides* extract. This test was conducted as previously described by Mizuki et al. [55]. Briefly, each mouse was individually suspended 50 cm above the floor by fixing the tail with black adhesive tape placed approximately 2 cm from the tip of the tail. This short term inescapable situation led to the development of an immobile posture indicating the hopeless behavior. The animal behavior was video-recorded for 6 min. The total immobility time was examined during the last 4-min period.

#### 4.8.3. Locomotor Activity Test

In order to exclude the false positive results from hopeless behavioral tests, locomotor activity test was conducted by evaluation of mice movement. The Y-Maze task was used to determine the locomotor activity. The Y-maze consisted of three arms 40 cm long, 18 cm high, 3 cm wide at the bottom, and 12 cm wide at the top, which were positioned at equal angles. One hour after drug administration, the animals were individually placed on one arm, and the total arm entries were recorded manually over an 8-min period for examining the locomotor activity [56].

### 4.9. Serum Corticosterone Level

Serum corticosterone level was determined by corticosterone (CORT) ELISA kit (Assaypro LLC. St. Charles, MO, USA) and corticosterone was used as a standard, according to the previously described procedure [57]. The serum of the sample 25 µL was added in 96-well microplates of ELISA kit and immediately added 25 µL of biotinylated corticosterone to each well. The 96-well microplates were gently mixed and incubated for 2 h at room temperature and then washed with 200 µL of wash buffer for five times and added 50 µL of streptavidin-peroxidase conjugate into each well before incubation for 30 minutes. Each well was washed with 200 µL of wash buffer for five times again and then added 50 µL of chromogen substrate and incubated for 20 minutes. The reaction was stopped by adding 50 µL of stop solution. The absorbance was immediately measured at 450 nm. 

### 4.10. Semi-Quantitative Reverse Transcription-Polymerase Chain Reaction (RT-PCR)

After completing the behavioral analysis, mice were decapitated under anesthesia. Mouse cyclic AMP response element binding protein (CREB), brain-derived neurotrophic factor (BDNF), and β-actin mRNAs expression in hippocampus and frontal cortex were semi-quantified by RT-PCR as described previously [44,58]. Total RNA was extracted from the tissues with TRIzol^®^ (Thermo Fisher Scientific Inc., CA, USA) according to the manufacturer’s instructions. First-strand cDNA was synthesized with oligo (dT) primers and SuperScript III reverse transcriptase (Thermo Fischer Scientific Inc., California, USA). PCR amplification was carried out using gene-specific PCR primer sets as follows: β-actin: 5′-AAC GGT CTC ACG TCA GTG TA-3′ (sense) and 5′-GTG ACA GCA TTG CTT CTG TG-3′ (antisense); BDNF: 5′-GAC AAG GCA ACT TGG CCT AC-3′ (sense) and 5′-CCT GTC ACA CAC GCT CAG CTC-3′ (antisense); and CREB: 5′-GAG GCA GCT TGA ACA ACA AC-3′ (sense) and 5′-TAC CCA GGG AGG AGC AAT AC-3′ (antisense). The PCR conditions consisted of 95 °C for 2 min, followed by 32 cycles of denaturation at 95 °C for 30 s, annealing at 60 °C for 60 s and extension at 72 °C for 60 s, with a final 5 min elongation at 72 °C. After the reaction is finished, in order to stain the PCR product 2 µL of Novel juice^®^ (GeneDireX Inc., Taoyuan, Taiwan) was added and separated by 38% acrylamide and 2% *bis*-acrylamide gel electrophoresis. The amplified products were visualized, photographed under ultraviolet and semi-quantified by GeneSnap software (GSL Biotech, Chicago, IL, USA) and ImageJ software (National Institutes of Health, Bethesda, MD, USA). The relative mRNA expression (BDNF/β-actin and CREB/β-actin) in each treatment were calculated by comparing with the sham group.

### 4.11. Human MAO-A and -B Inhibitory Assay

The MAOs inhibitory activity assay of the *A. philoxeroides* extract and some of its flavonoid constituents, using recombinant human MAO-A and MAO-B (Sigma-Aldrich, St. Louis, MO, USA) as enzyme sources, was performed according to the method described by Manley-King et al. [15]. Briefly, the crude extract 100 mg was dissolved in 400 µL DMSO to prepare 250 mg/mL as a stock solution. Serial dilution of test samples (20 µL) for each concentration was used to test the activity. For **1a**, **1b**, 3a, **3b** and **5**, a stock solution of 1000 μM in DMSO was prepared and a serial dilution of the test sample (20 μL) of each concentration was used to test the activity. Concentration of MAOs was 0.0075 mg/mL for MAO-A inhibitor test, 9 µL of kynuramine (Sigma-Aldrich, St. Louis, MO, USA) was mixed with 469.5 µL of potassium phosphate buffer (pH 7.4). For MAO-B inhibitor test, 6 µL of kynuramine was mixed with 472.5 µL of potassium phosphate buffer (pH 7.4). Then 1.5 µL of the enzymes were added before incubation at 37 °C for 20 min. The reaction was stopped by adding 400 µL of 2N NaOH and 1000 µL of water. Measurement of 4-hydroxyquinoline (Sigma-Aldrich, St. Louis, MO, USA) was carried out by fluorescence spectrophotometry with the excitation wavelength at 310 nm and the emission wavelength at 400 nm. The potencies of the inhibition test were calculated from experimental results sigmoidal dose-response curves and expressed as IC_50_ values which were determined in duplicate and expressed as mean ± standard deviation (SD). Data were analyzed by Prism 5 software package (GraphPad Software, version 5, San Diego, CA, USA). The IC_50_ values were converted to the corresponding *Ki* values according to the equation *Ki* = IC50/(1+[S]/Km). Clorgyline (Sigma-Aldrich, MO, USA) and deprenyl (Sigma-Aldrich, St. Louis, MO, USA) were used as control for selective MAO-A and MAO-B inhibitors, respectively.

### 4.12. Statistical Analysis

The data obtained from *in vitro* studies were expressed as the mean ± SD. Behavioral and neurochemical data were expressed as the mean ± S.E.M. and were examined by paired Student's t-test for sham versus OVX group or one-way analysis of variance (ANOVA) followed by *post hoc* Tukey test for multiple comparisons. Differences of *p* < 0.05 were considered to be statistically significant. The analysis was conducted using SigmaStat^®^ ver. 3.5 (SYSTAT Software Inc., San Jose, CA, USA).

## 5. Conclusions

*Alternanthera philoxeroides* (Mart.) Griseb and *Alternanthera sessilis* (L.) R. Br. *ex* DC. are used indiscriminately as ingredients of a decoction in Thai folk medicine for stimulating milk secretion and for treatment of postpartum depression. However, there was no evidence-based study of *A. philoxeroides* for the claimed effects. By using ovariectomized mouse model we have proved through the forced swimming and tail suspension tests that the crude ethanol extract of this plant ameliorated the depressant-like behaviors of the estrogen-deprived mice. Moreover, the crude extract was found to recover the weight and volume of the uterus of the OVX mice as well as to up-regulate the expression of the BDNF mRNA in the hippocampus and frontal cortex similar to 17β-estradiol, a drug used for ERT. It is legitimate to assume that these activities could be, in part, due to the flavonoid constituents of *A. philoxeroides*. Another mechanism that can contribute to the antidepressant effects of both extract and flavonoids, isolated from *A. philoxeroides*, is their intervention in MAOs function. Thus, this study contributes to a scientifically based-evidence for the use of *A. philoxeroides* in Thai folk medicine.

## Figures and Tables

**Table 1:** Estrogen level, weight and volume of uterus of OVX mice treated with the ethanol crude extract of *A. phliroxeroides* (AP) and 17β-estradiol (E2).

Group	17β-estradiol (pg/mL)	Uterine Weight (g/kg)	Uterine Volume (cm^3^/kg)
Sham	17.91 ± 1.10 **	4.105 ± 0.01 **	2.500 ± 0.03 **
OVX Vehicle-treated	8.27 ± 0.32	1.675 ± 0.01	0.500 ± 0.00
OVX + E_2_ (1 μg/kg/day)	24.81 ± 1.11 **	3.055 ± 0.04 **	2.305 ± 0.02 **
OVX + AP (250 mg/kg/day)	12.24 ± 0.52 **	2.600 ± 0.01 *	1.143 ± 0.02 **
OVX + AP (500 mg/kg/day)	13.19 ± 0.01 **	3.029 ± 0.01 *	1.714 ± 0.04 **

Data were expressed as mean ± SEM (Serum of estrogen, *n* = 3–4; Uterine weight and volume, *n* = 8–12). All post hoc comparisons were vs. vehicle-treated OVX group by one-way ANOVA. ** p < 0.001 and * p < 0.05.

**Table 2:** Inhibitory effect of the ethanol crude extract of *A. philoxeroides* and **1a**, **1b**, **3a**, **3b** and **5** on monoamine oxidase (MAO)-A and MAO-B.

	IC_50_ (μM)		*Ki* (μM)		Si	
Extract/compound	MAO-A	MAO-B	MAO-A	MAO-B	MAO-A	MAO-B
Extract of *A. philoxeroides* (μg/mL)	252.9 ± 0.02	90.69 ± 0.02	66.640	39.0640	1.7050	0.5860
**1a**	0.00046 ± 0.04	0.00060 ± 0.12	0.00012	0.00026	0.4681	2.1364
**1b**	0.00206 ± 0.04	0.00022 ± 0.12	0.00054	0.00010	5.6681	0.1764
**3a**	18.37 ± 1.47	0.6748 ± 0.46	4.8405	0.2900	16.6534	0.0601
**3b**	0.0541 ± 0.01	0.1293 ± 0.42	0.1328	0.0557	2.3850	0.4193
**5**	3.051 ± 0.35	0.5441 ± 0.33	0.8039	0.2344	3.4303	0.2915
Clorgyline *	0.0016 ± 0.05	0.4130 ± 0.08	0.0004	0.1779	0.0024	421.950
Deprenyl **	6.579 ± 0.05	2.024 ± 0.04	1.7336	0.8718	1.9885	0.5029

All data are expressed as mean ± SD. * Clorgyline is a reference for selective of MAO-A inhibitor, ** Deprenyl is a reference for selective of MAO-B inhibitor. *Ki* is the corresponding enzyme-inhibitor dissociation constant and Si is the selectivity index between MAO-A and MAO-B.

**Table 3:** ^1^H- and ^13^C-NMR (DMSO-*d*_6_, 500.13 and 125.4 MHz) and HMBC assignment for **2**.

Position	δ_C_, Type	δ_H_, (*J* in Hz)	COSY	HMBC
2	164.1, C	-		
3	102.8, CH	6.70, s		C-2, 4, 4a, 1′
4	181.9, CO	-		
4a	103.0, C *	-		
5	157.5, C	-		
6	110.1, C	-		
7	162.0, C	-		
8	94.6, CH	6.52, s		C-4a, 6, 7, 8a
8a	156.1, C	-		
1′	121.5, C *	-		
2′	113.4, CH	7.41, d (2.2)	H-6′	C-2, 3′, 4′
3′	145.8, C	-		
4′	150.0, C	-		
5′	116.0, CH	6.89, d (8.4)	H-6′	C-1′, 3′, 4′
6′	119.1, CH	7.43, dd (8.4, 2.2)	H-2′, 5′	C-2, 2′, 4′
1″	70.1, CH	4.99, dd (11.9, 2.9	H-2″	
2″	32.4, CH_2_	2.05, q (11.7)	H-1″	
		1.60, m	H-1″	
3″	68.5, CH	3.71, m	H-4″	
4″	69.6, CH	3.45 **	H-3″	
5″	74.4, CH	3.65, q (6.4)	H-6″	
6″	1.18, CH_3_	1.18, d (6.4)	H-5″	C-4″, 5″
OH-5	-	13.53, s		

* observed in HMBC spectrum, ** under the water peak.

**Table 4:** ^1^H- and ^13^C-NMR (DMSO-*d*_6_, 500.13 and 125.4 MHz) and HMBC assignment for **3a**.

Position	δ_C_, Type	δ_H_, (*J* in Hz)	COSY	HMBC	NOESY
2	163.7, C	-	-		
3	103.1, CH	6.96, s		C-4, 4a	
4	181.4, CO	-			
4a	1028, C	-			
5	161.8, C	-			
6	106.0, C	-			
7	163.6, C	-			
8	94.0, CH	6.55, s		C-4a, 6, 8a	
8a	157.4, C	-			
1′	121.3, C	-			
2′	110.2, CH	7.57, brs		C-4′, 6′	OMe
3′	148.1, C	-			
4′	150.9, C	-			
5′	115.8, CH	6.95, d (8.2)		C-1′, 2′, 3′, 4′	
6′	120.5, CH	7.58, dd (8.2, 2.1)		C-2, 2′, 4′	
1″	134.0, CH	7.91, d (16.2)		2″, 3″, 5, 7	
2″	119.6, CH	6.86, d (16.2)		3″, 6	
3″	168.8, CO	-			
OMe-3′	56.0, CH_3_	3.89, s		C-3′	H-2′
OH-5	-	14.45, s		C-4a, 5, 6	
OH-3″	-	10.06, br			

## Supplementary Materials

The following are available online, Figure S1: Structures of indole-3-carbaldehyde, oleanonic acid, vanillic acid, *p*-hydroxybenzoic acid, isolated from the crude ethanol extract of *Alternanthera philoxeroides* (Mart.) Griseb, Figures S2–S29: 1D and 2D-NMR spectra of isolated compounds, Figure S30: Ortep view of althernanthin (**1a**).

## References

[1] Fang J.B., Yao Z., Chen J.C., Liu Y.W., Takaishi Y., Duan H.Q. (2009). Cytotoxic triterpene saponins from *Alternanthera philoxeroides*. J. Asian Nat. Prod. Res..

[2] Purkayastha J., Nath S.C., Islam M. (2005). Ethnobotany of medicinal plants from Dibru-Saikhowa Biosphere Reserve of Northeast India. Fitoterapia.

[3] Rahman Rahman A.H.M., Gulshana M.I.A. (2014). Taxonomy and medicinal uses on Amaranthaceae family of Rajshahi, Bangladesh. Appl. Ecol. Environ. Sci..

[4] Sunmathi D., Sivakumar R., Ravikumar K. (2016). *In vitro* Anti-inflammatory and antiarthritic activity of ethanolic leaf extract of *Alternanthera sessilis* (L.) R.BR. ex DC and *Alternanthera philoxeroides* (Mart.) Griseb. Int. J. Adv. Pharm. Biol. Chem..

[5] Rattanathongkom A., Lee J.B., Hayashi K., Sripanidkulchai B.O., Kanchanapoom T., Hayashi T. (2009). Evaluation of chikusetsusaponin IVa isolated from *Alternanthera philoxeroides* for its potency against viral replication. Planta Med..

[6] Zhou B.N., Blasko G., Cordell G.A. (1988). Althernabthin, a C-glycosylated flavonoid from *Althernanthera philoxeroides*. Phytochemistry.

[7] Bhattacherjee A., Ghosh I., Sil R., Datta A. (2014). Isolation and characterisation of methanol-soluble fraction of *Alternanthera philoxeroides* (Mart.)–evaluation of their antioxidant, α-glucosidase inhibitory and antimicrobial activity *in vitro* system. Nat. Prod. Res..

[8] Ashour M.A., Elkhayat E.S., Ebel R., Edrada R., Proksch P. (2007). Indole alkaloid from the Red Sea sponge *Hyrtios erectus*. Arkivoc.

[9] Campos A.M., Oliveira F.S., Iracema M., Machado L., Braz-filho R., Matos F.J.A. (1991). Triterpenes from *Cedrela odorata*. Phytochemistry.

[10] Kitanaka S., Takido M. (1992). Demethyltorosaflavones C and D from *Cassia nomame*. Phytochemistry.

[11] Zhao J., Pawar R.S., Ali Z., Khan I.A. (2007). Phytochemical investigation of *Turnera. diffusa*. J. Nat. Prod..

[12] Chou C.J., Wang C.B., Lin L.C. (1976). Chrysoeriol 7-*O*-rhamnoside from *Sedum formosanum*. Phytochemistry.

[13] Monthakantirat O., De-Eknamkul W., Umehara K., Yoshinaga Y., Toshio Miyase T., Warashina T., Noguchi H. (2005). Phenolic constituents of the rhizomes of the Thai medicinal plant *Belamcanda chinensis* with proliferative activity for two breast cancer cell lines. J. Nat. Prod..

[14] Cheng H.C. (2001). The power issue: Determination of *K*_B_ or *K*_i_ from IC_50_: A closer look at the Cheng–Prusoff equation, the Schild plot and related power equations. J. Pharmacol. Toxicol. Methods.

[15] Manley-King C.I., Bergh J.J., Petzer J.P. (2011). Inhibition of monoamine oxidase by C5-substituted phthalimide analogues. Bioorg. Med. Chem..

[16] Bao A.M., Ji Y.F., Van Someren E.J., Hofman M.A., Liu R.Y., Zhou J.N. (2004). Diurnal rhythms of free estradiol and cortisol during the normal menstrual cycle in women with major depression. Horm. Behav..

[17] Heydarpour P., Salehi-Sadaghiani M., Javadi-Paydar M., Rahimian R., Fakhfouri G., Khosravi M., Khoshkish S., Gharedaghi M.H., Ghasemi M., Dehpour A.R. (2013). Estradiol reduces depressive-like behavior through inhibiting nitric oxide/cyclic GMP pathway in ovariectomized mice. Horm. Behav..

[18] Rasgon N.L., Altshuler L.L., Fairbanks L.A., Dunkin J.J., Davtyan C., Elman S., Rapkin A.J. (2002). Estrogen replacement therapy in the treatment of major depressive disorder in perimenopausal women. J. Clin. Psychiatry.

[19] Lagunas N., Calmarza-Font I., Diz-Chaves Y., Garcia-Segura L.M. (2010). Long-term ovariectomy enhances anxiety and depressive-like behaviors in mice submitted to chronic unpredictable stress. Horm. Behav..

[20] Estrada-Camarena E., Rivera N.M., Berlanga C., Fernández-Guasti A. (2008). Reduction in the latency of action of antidepressants by 17 beta-estradiol in the forced swimming test. Psychopharmacology.

[21] Dhir A., Kulkarni S.K. (2008). Antidepressant-like effect of 17β-estradiol: Involvement of dopaminergic, serotonergic, and (or) sigma-1 receptor systems. Can. J. Physiol. Pharmacol..

[22] Kudielka B.M., Hellhammer D.H., Wüst S. (2009). Why do we respond so differently? Reviewing determinants of human salivary cortisol responses to challenge. Psychoneuroendocrinology.

[23] Scharfman H.E., MacLusky N.J. (2006). Estrogen and brain-derived neurotrophic factor (BDNF) in hippocampus: Complexity of steroid hormone-growth factor interactions in the adult CNS. Front. Neuroendocrinol..

[24] Zhang L., Xiong W., Xiong Y., Liu H., Liu Y. (2016). 17 β-Estradiol promotes vascular endothelial growth factor expression via the Wnt/β-catenin pathway during the pathogenesis of endometriosis. Mol. Hum. Reprod..

[25] Clark-Raymond A., Meresh E., Hoppensteadt D., Fareed J., Sinacore J., Halaris A. (2014). Vascular endothelial growth factor: A potential diagnostic biomarker for major depression. J. Psychiatr. Res..

[26] Gibaldi M. (2000). Are phytoestrogens a “natural alternative” to estrogen replacement therapy?. West. J. Med..

[27] Aidelsburger P., Schauer S., Grabein K., Wasem J. (2012). Alternative methods for the treatment of post-menopausal troubles. GMS Health Technol. Assess..

[28] Miksicek R.J. (1993). Commonly occurring plant flavonoids have estrogenic activity. Mol. Pharmacol..

[29] Loc kridge A., Su J., Yuan L.L. (2010). Abnormal 5-HT modulation of stress behaviors in the Kv4.2 knoc kout mouse. Neuroscience.

[30] Suetsugi M., Su L., Karlsberg K., Yuan Y.C., Chen S. (2003). Flavone and isoflavone phytoestrogens are agonists of estrogen-related receptors. Mol. Cancer Res..

[31] Kitanaka S., Ogata K., Takido M. (1989). Studies on the constituents of the leaves of *Cassia torosa* Cav. I. The structure of two new C-glycosylflavones. Chem. Pharm. Bull..

[32] Kitanaka S., Takido M. (1991). Studies on the constituents of the leaves of *Cassia torosa* CaV. II. The structure of two novel flavones torosaflavone C and D. Chem. Pharm. Bull..

[33] Llewellyn S., Fitzpatrick R., Kenny D.A., Patton J., Wathes D.C. (2008). Endometrial expression of the insulin-like growth factor system during uterine involution in the postpartum dairy cow. Domest. Anim. Endocrinol..

[34] Christoffel J., Rimoldi G., Wuttke W. (2006). Effects of 8-prenylnaringenin on the hypothalamo-pituitary-uterine axis in rats after 3-month treatment. J. Endocrinol..

[35] Deshaies Y., Dagnault A., Lalonde J., Richard D. (1997). Interaction of corticosterone and gonadal steroids on lipid deposition in the female rat. Am. J. Physiol. Endocrinol. Metab..

[36] Shimizu H., Ohtani K., Kato Y., Yoshito Tanaka Y., Mori M. (1996). Estrogen increases hypothalamic neuropeptide Y (NPY) mRNA expression in ovariectomized obese rat. Neurosci. Lett..

[37] Varghese F.P., Brown E.S. (2001). The Hypothalamic-Pituitary-Adrenal Axis in Major Depressive Disorder: A Brief Primer for Primary Care Physicians. Prim. Care Companion. J. Clin. Psychiatry.

[38] Jones A.B., Gupton R., Curtis K.S. (2016). Estrogen and voluntary exercise interact to attenuate stress-induced corticosterone release but not anxiety-like behaviors in female rats. Behav. Brain Res..

[39] An L., Zhang Y.Z., Liu X.M., Yu N.J., Chen H.X., Zhao N., Yuan L., Li Y.F. (2011). Total Flavonoids Extracted from Xiaobuxin-Tang on the Hyperactivity of Hypothalamic-Pituitary-Adrenal Axis in Chronically Stressed Rats. Evid. Based Complement. Alternat. Med..

[40] Henderson V.W., Brinton R.D. (2010). Menopause and mitochondria: Windows into estrogen effects on Alzheimer's disease risk and therapy. Prog. Brain Res..

[41] Xiao L., Shu C., Tang J., Wang H., Liu Z., Wang G. (2011). Effects of different CMS on behaviors, BDNF/CREB/Bcl-2 expression in rat hippocampus. Biomed. Aging Pathol..

[42] Bathina S., Das U.N. (2015). Brain-derived neurotrophic factor and its clinical implications. Arch. Med. Sci..

[43] Sohrabji F., Miranda R.C., Toran-Allerand C.D. (1995). Identification of a putative estrogen response element in the gene encoding brain-derived neurotrophic factor. Proc. Natl. Acad. Sci. USA.

[44] Monthakantirat O., Sukano W., Umehara K., Noguchi H., Chulikhit Y., Matsumoto K. (2014). Effect of miroestrol on ovariectomy-induced cognitive impairment and lipid peroxidation in mouse brain. Phytomed. Int. J. Phytother. Phytopharm..

[45] Neshatdoust S., Saunders C., Castle S.M., Vauzour D., Williams C., Butler L., Lovegrove J.A., Spencer J.P.E. (2016). High-flavonoid intake induces cognitive improvements linked to changes in serum brain-derived neurotrophic factor: Two randomised, controlled trials. Nutr. Healthy Aging.

[46] Numakawa T. (2014). Possible protective action of neurotrophic factors and natural compounds against common neurodegenerative diseases. Neural Regen. Res..

[47] Schaaf M.J.M., de Kloet E.R., Vreugdenhil E. (2000). Corticosterone effects on BDNF expression in the hippoc ampus. Stress.

[48] Patil P.O., Bari S.B., Firke S.D., Deshmukh P.K., Donda S.T., Patil D.A. (2013). A comprehensive review on synthesis and designing aspects of coumarin derivatives as monoamine oxidase inhibitors for depression and Alzheimer’s disease. Bioorg. Med. Chem..

[49] Bennett D.A., Phun L., Polk J.F., Voglino S.A., Zlotnik V., Raffa R.B. (1998). Neuropharmacology of St. John’s Wort (Hypericum). Ann. Pharmacother..

[50] Larit F., Elokely K.M., Chaurasiya N.D., Benyahia S., Nael M.A., León F., Abu-Darwish M.S., Efferth T., Wang Y.H., Belouahem-Abed D. (2018). Inhibition of human monoamine oxidase A and B by flavonoids isolated from two Algerian medicinal plants. Phytomedicine.

[51] Carradori S., D’Ascenzio M., Chimenti P., Secci D., Bolasco A. (2014). Selective MAO-B inhibitors: A lesson from natural product. Mol. Divers..

[52] Carradori S., Gidaro MC., Petzer A., Costa G., Guglielmi P., Chimenti P., Alcaro S., Petzer J.P. (2016). Inhibition of human monoamine oxidase: Biological and molecular modeling studies on selected natural flavonoids. J. Agric. Food Chem..

[53] Parasuraman S., Raveendran R., Kesavan R. (2010). Blood sample collection in small laboratory animals. J. Pharmacol. Pharmacother..

[54] Vogel G., Vogel W.H., Vogel H.G. (2007). Psychotropic and Neurotropic Activity. Drug Discovery and Evaluation: Pharmacological Assays.

[55] Mao X., Liao Z., Guo L., Xu X., Wu B., Xu M., Zhao X., Bi K., Jia Y. (2015). Schisandrin C ameliorates learning and memory deficits by Aβ1–42-induced oxidative stress and neurotoxicity in mice. Phytother. Res..

[56] Mizuki D., Qi Z., Tanaka K., Fujiwara H., Ishikawa T., Higuchi Y., Matsumoto K. (2014). *Butea superba*–induced amelioration of cognitive and emotional deficits in olfactory bulbectomized mice and putative mechanisms underlying its actions. J. Pharmacol. Sci..

[57] Mizuki D., Matsumoto K., Tanaka K., Le X.Y., Fujiwara H., Ishikawa T., Higuchi Y. (2014). Antidepressant-like effect of *Butea superba* in mice exposed to chronic mild stress and its possible mechanism of action. J. Ethnopharmacol..

[58] Carroll P., Casimir D., Barlett J.M.S., Stirling D. (2003). PCR Patent Issues. PCR Protocols, Methods in Molecular Biology^TM^.

