# Supplementary material for: Chemical Constituents and Antidepressant-Like Effects in Ovariectomized Mice of the Ethanol Extract of Alternanthera philoxeroides
Source: Molecules. 2018 Aug 31;23(9):2202. doi: 10.3390/molecules23092202 (PMC6225253; doi:10.3390/molecules23092202)

Supplementary Materials

Chemical Constituents and Antidepressant-like Effect in Ovariectomized Mice of the Ethanol Extract of *Alternanthera philoxeroides*

Charinya Khamphukdee [1], Orawan Monthakanthirat [2*], Yaowared Chulikit [2], Suradet Buttachon [3, 4], Michael Lee [5], Artur M. S. Silva [6], Nazim Sekeroglu [7] and Anake Kijjoa [3, 4*]

[1] Graduate School of Pharmaceutical Sciences, Khon Kaen University, Khon Kaen 40002, Thailand. E-mail: jarin_jd@yahoo.com.
[2] Division of Chemistry, Faculty of Pharmaceutical Sciences, Khon Kaen University, Khon Kaen 40002, Thailand. E-mail: oramon@kku.ac.th (O.M.); yaosum@kku.ac.th Y.Ch.).
[3] *ICBAS*-Instituto de Ciências Biomédicas Abel Salazar, Rua de Jorge Viterbo Ferreira, 228, 4050-313 Porto, Portugal.
[4] Interdisciplinary Centre of Marine and Environmental Research (CIIMAR), Terminal de Cruzeiros do Porto de Lexões, Av. General Norton de Matos s/n, 4450-208, Matosinhos, Portugal. E-mail: nokrari_209@hotmail.com.
[5] Department of Chemistry, University of Leicester, University Road, Leicester LE 7 RH, UK. E-mail: ml34@leicester.ac.uk.
[6] Departamento de Química & QOPNA, Universidade de Aveiro, 3810-193 Aveiro, Portugal. E-mail: artur.silva@ua.pt.
[7] Department of Food Engineering, Faculty of Architecture and Engineering, Kilis 7 Aralık University, 79000, Kilis, Turkey. E-mail: nsekeroglu@gmail.com.

Figure S1. Structures of indole-3-carbaldehyde, oleanonic acid, vanillic acid, *p*-hydroxybenzoic acid isolated from *Althernanthera philoxeroides* (Mart.) Griseb.

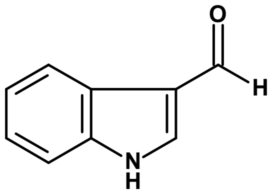

Indole-3-carbaldehyde

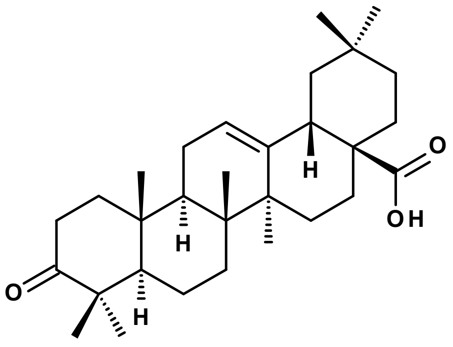

Oleanonic acid

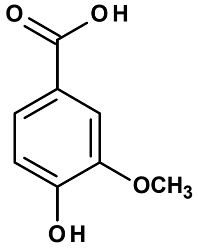

Vanillic acid

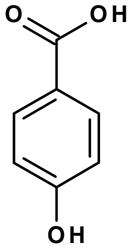

***p*-Hydroxybenzoic acid**

Figure S2. ^{1}H NMR spectrum of indole-3-carbaldehyde (DMSO-d_6, 500 MHz).

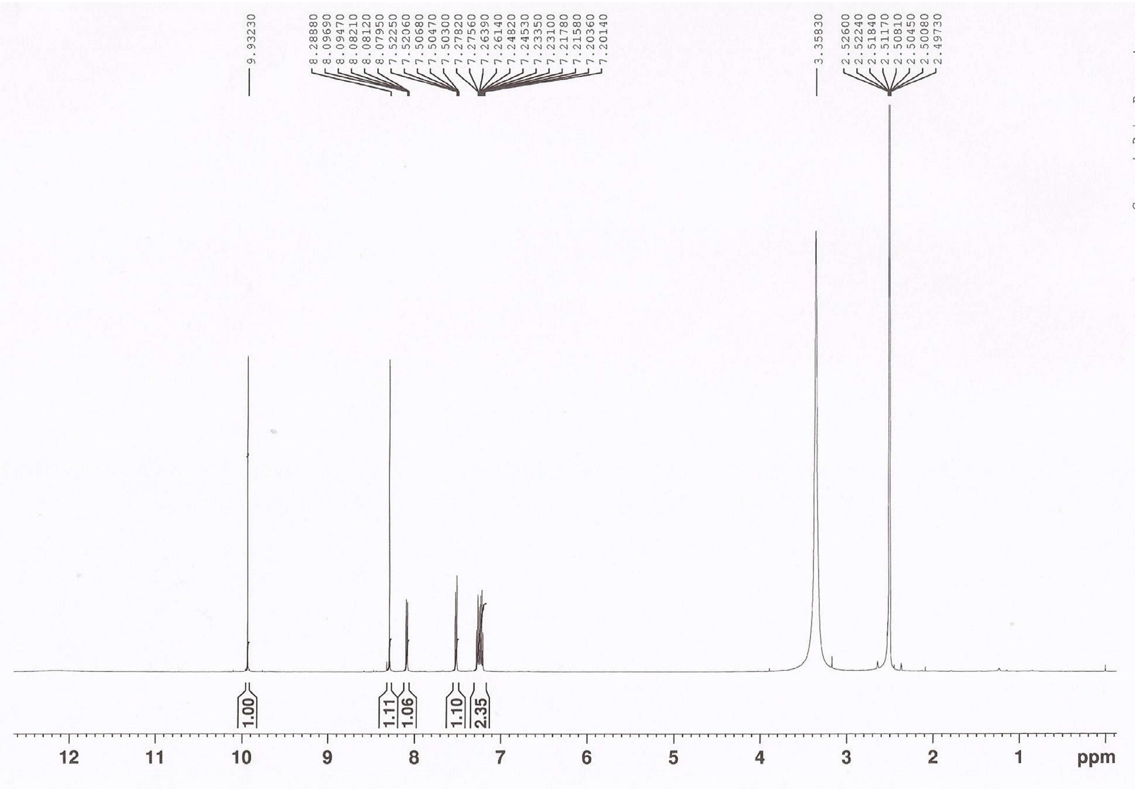

Figure S3. ^{13}C NMR spectrum of indole-3-carbaldehyde (DMSO-d_6, 125 MHz).

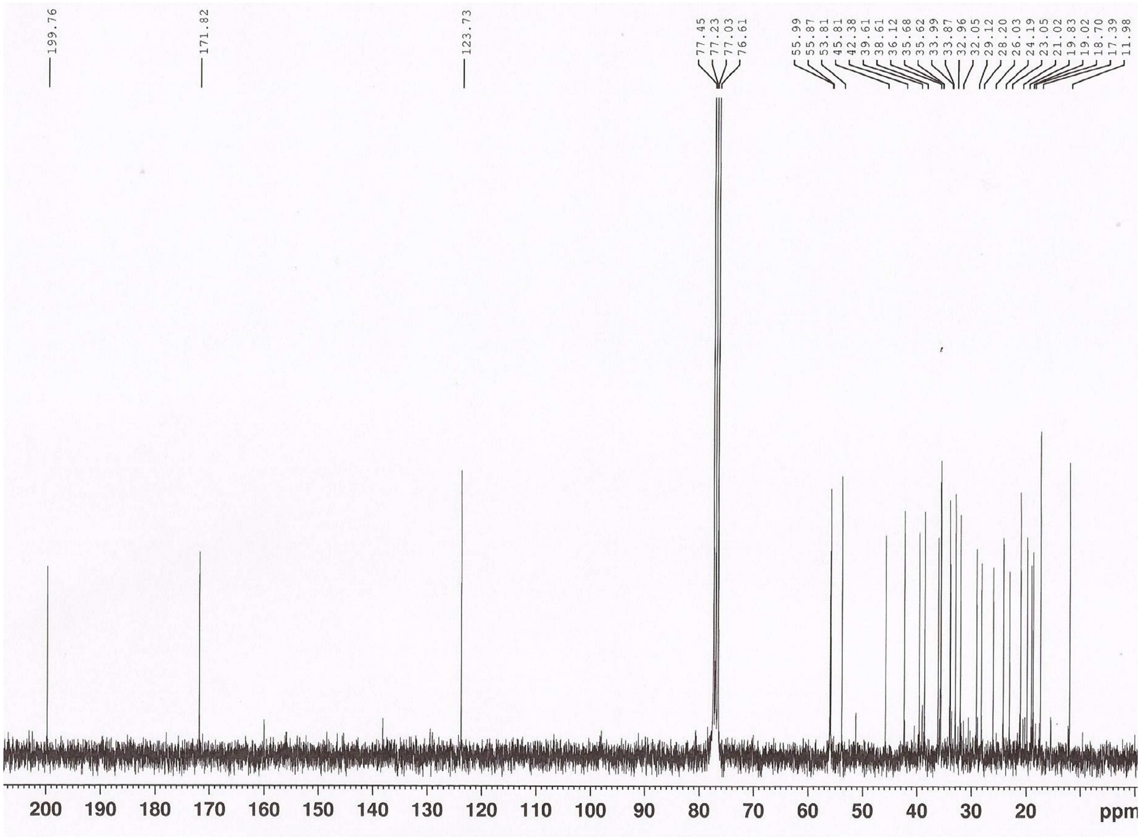

Figure S4. ^{1}H NMR spectrum of oleanonic acid ($CDCl_3$, 500 MHz).

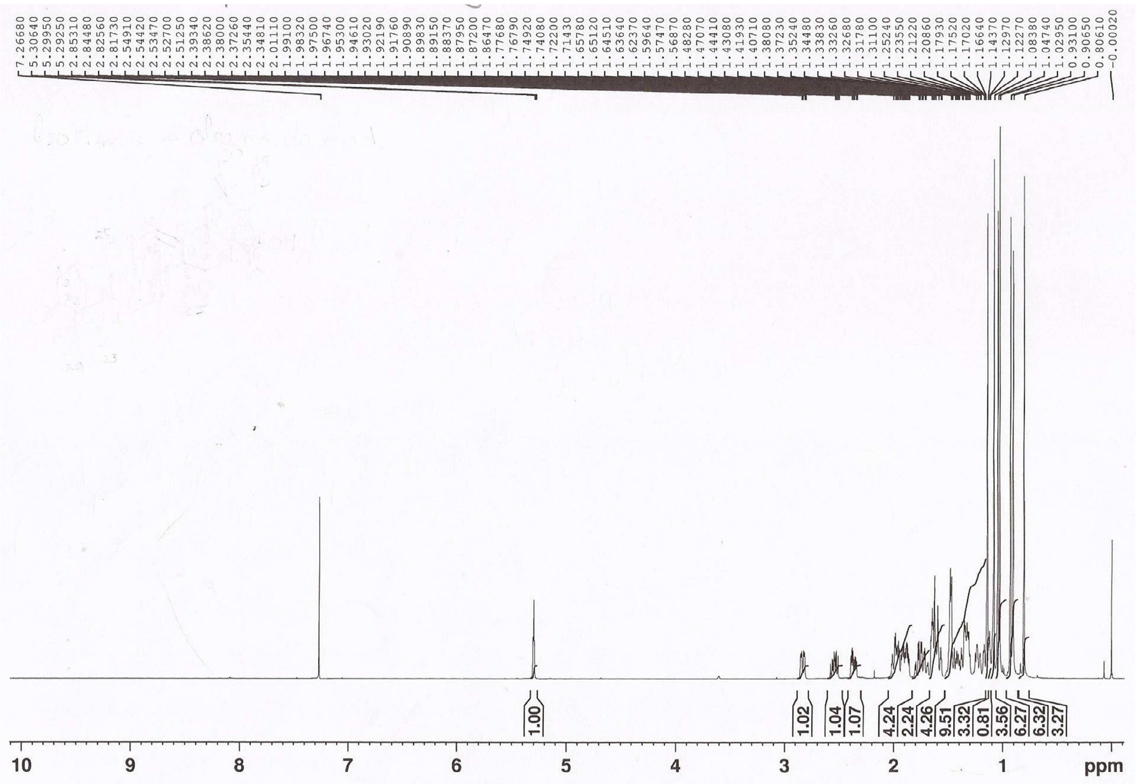

Figure S5. ^{13}C NMR spectrum of oleanonic acid ($CDCl_3$, 125 MHz).

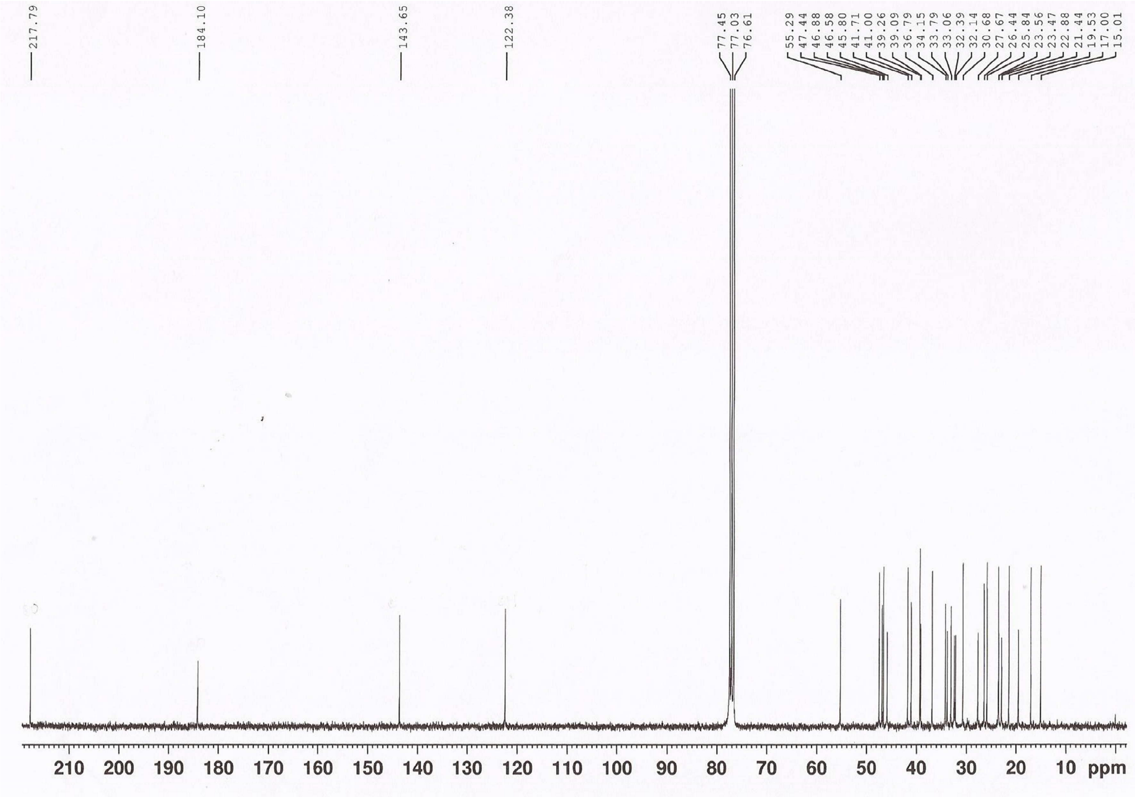

Figure S6. ^{1}H NMR spectrum of vanillic acid (DMSO-*d6*, 500 MHz).

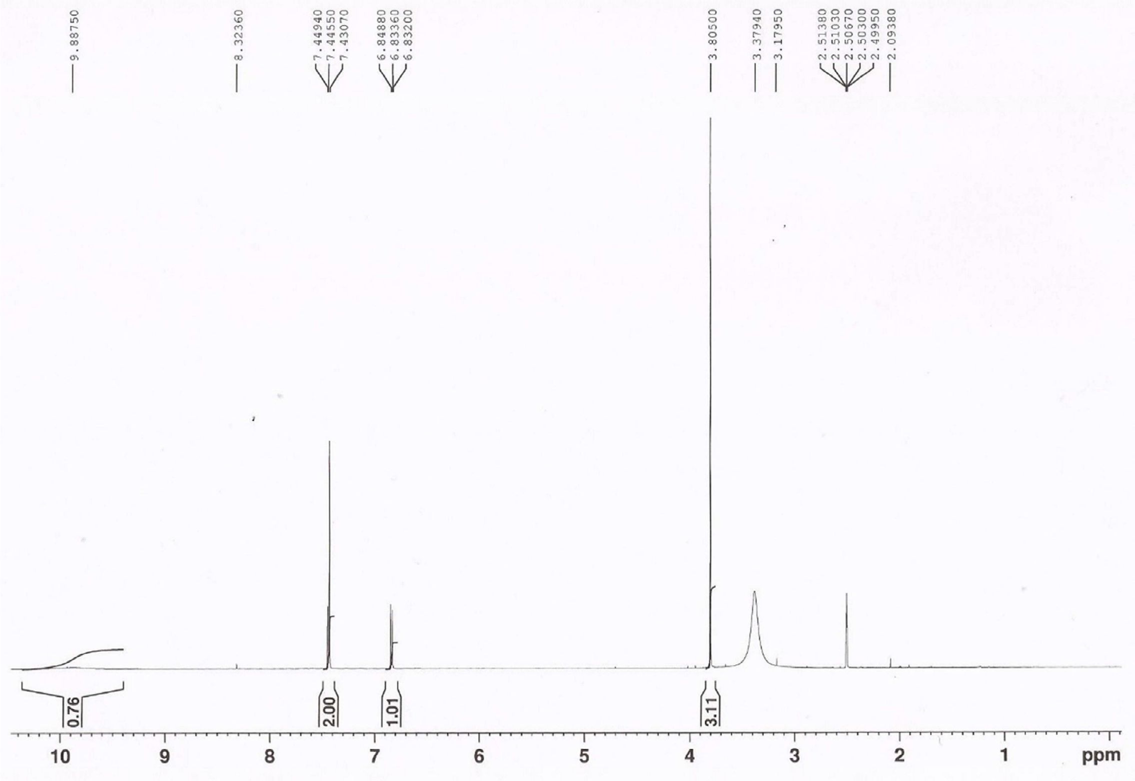

Figure S7. ^{13}C NMR spectrum o (DMSO-*d6*, 125 MHz).

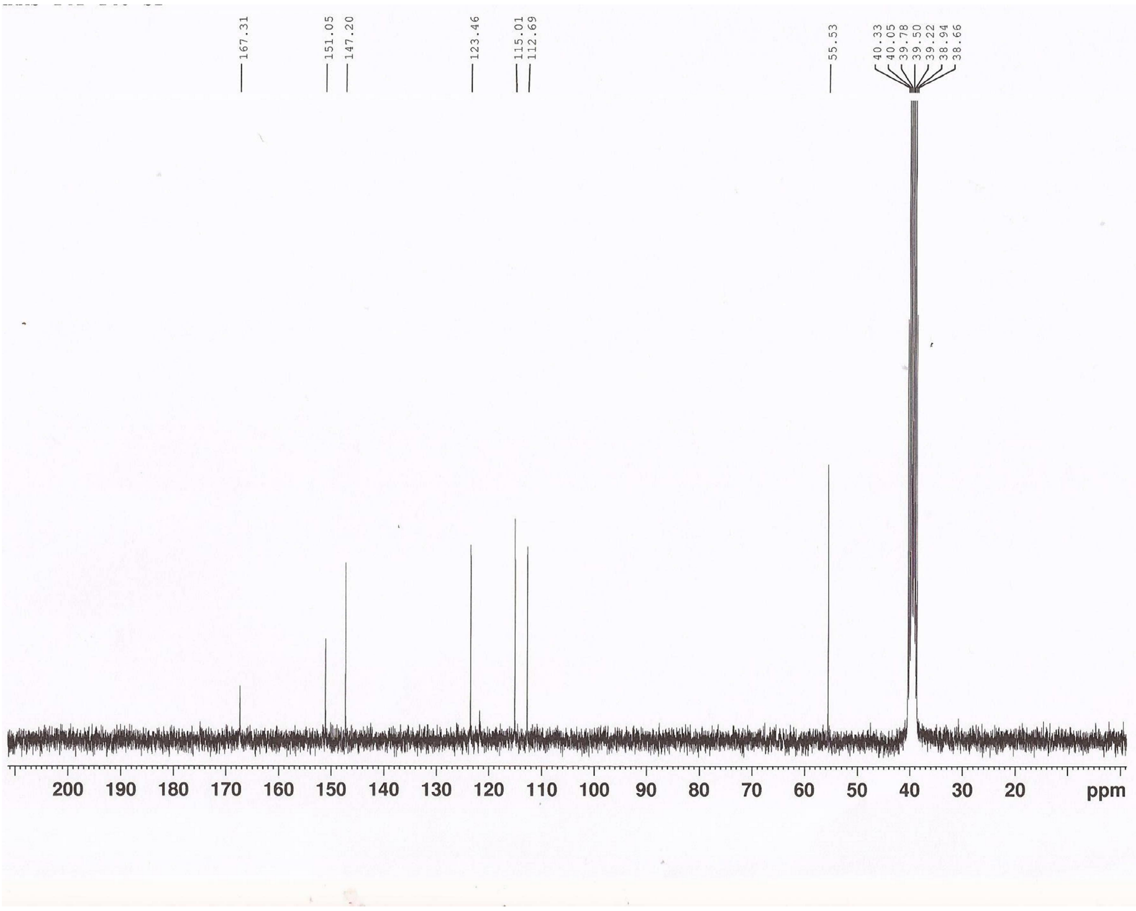

Figure S8. ^{1}H NMR spectrum of *p*-hydroxybenzoic acid (DMSO-*d6*, 500 MHz).

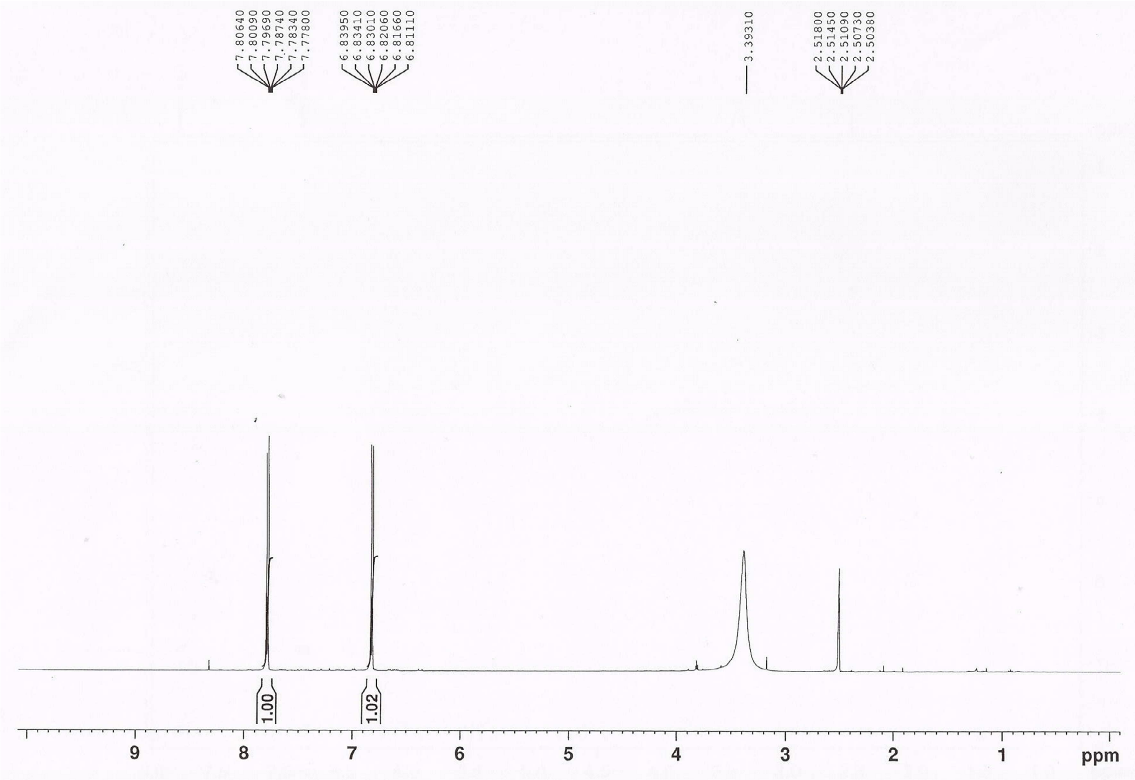

Figure S9. ^{13}C NMR spectrum of *p*-hydroxybenzoic acid (DMSO-*d6*, 125 MHz).

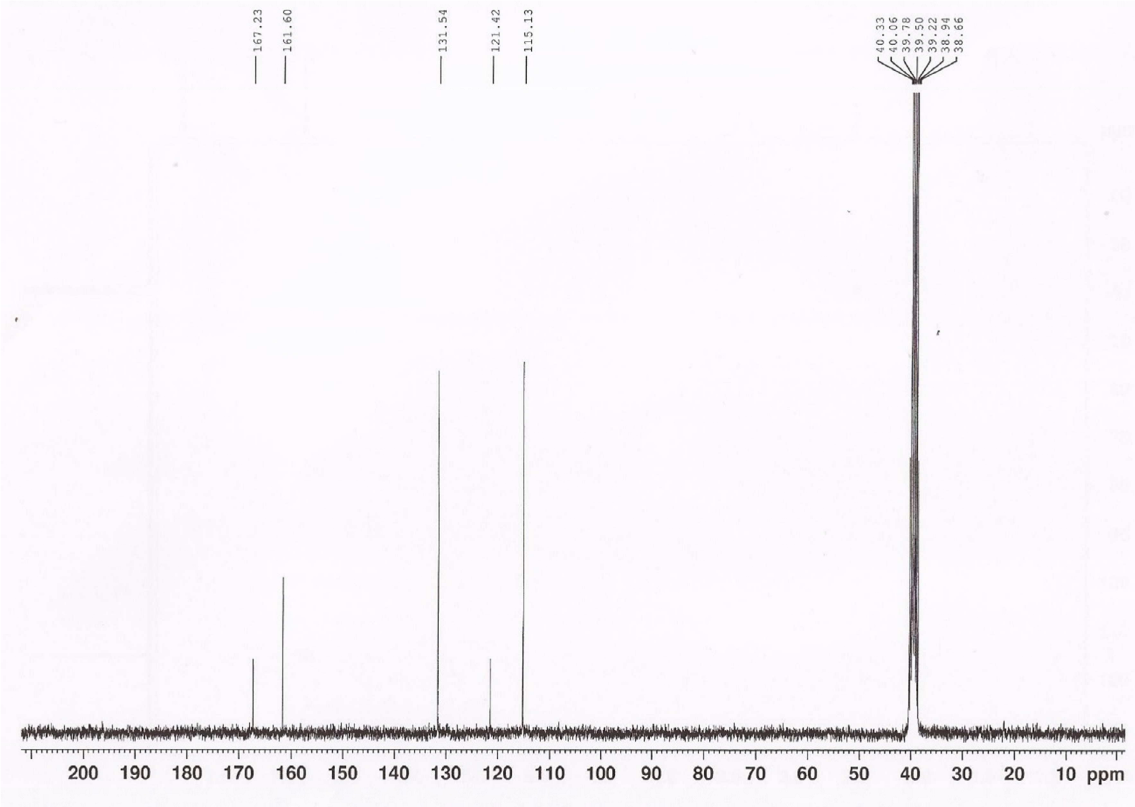

Figure S10. ^{1}H NMR spectrum of **1a** (DMSO-*d6*, 500.13 MHz).

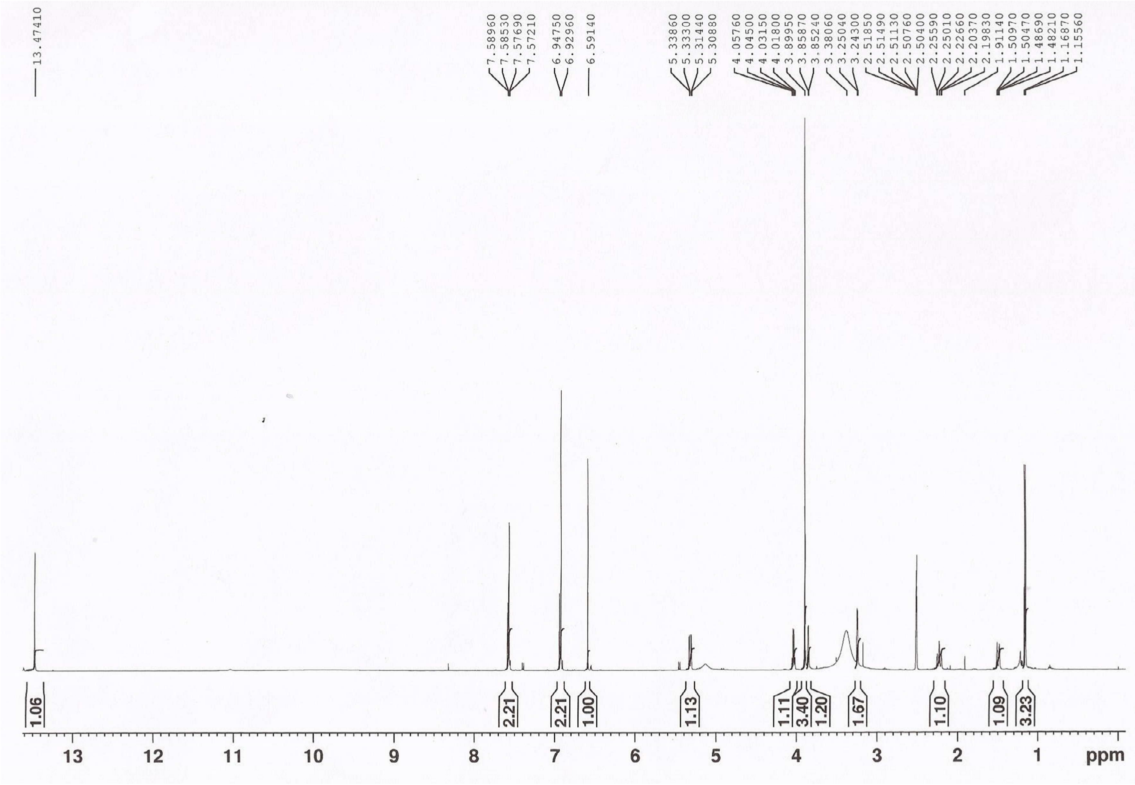

Figure S11. ^{13}C NMR spectrum of **1a**(DMSO-*d6*,, 125.4 MHz).

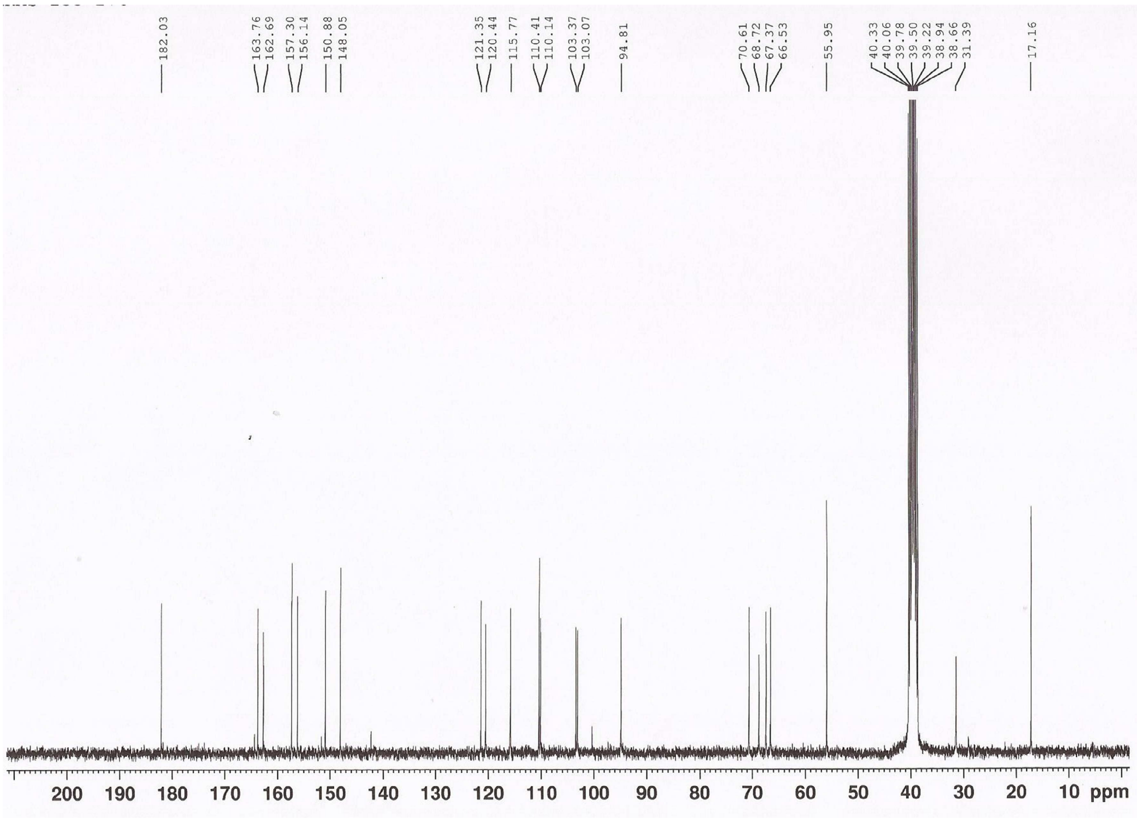

Figure S12. ^{1}H NMR spectrum of **1b** (DMSO-*d6*, 500.13 MHz).

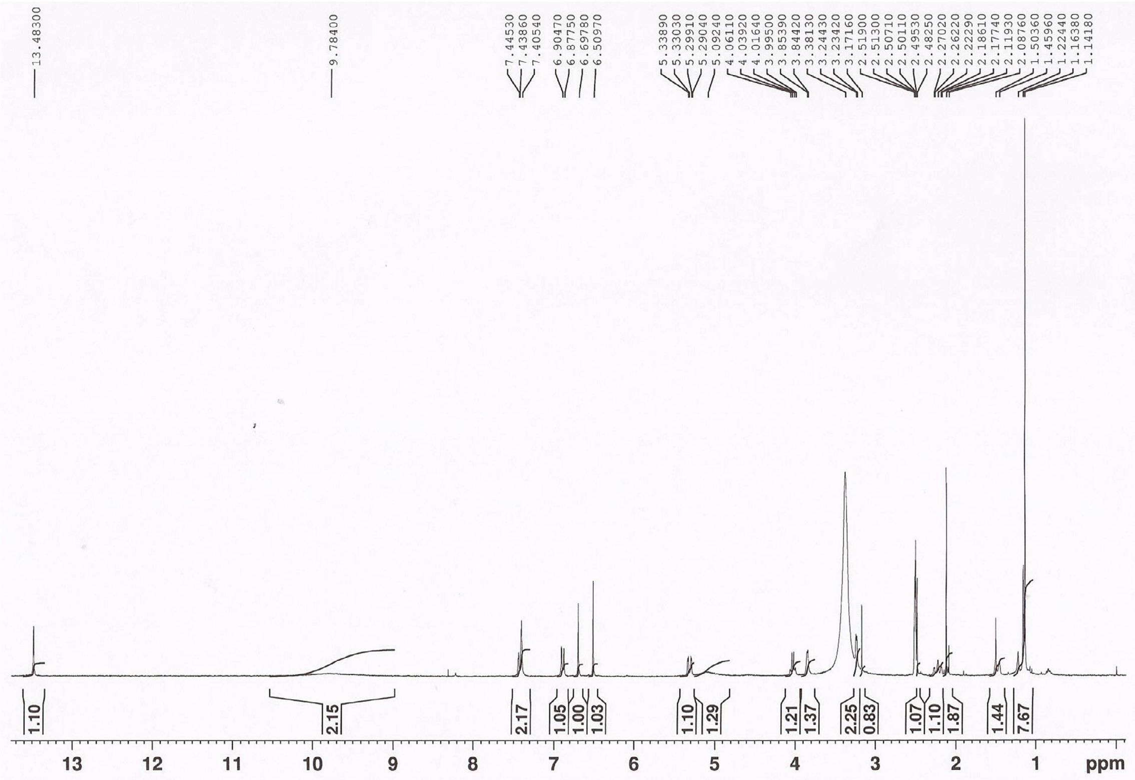

Figure S13. ^{13}C NMR spectrum of **1b** (DMSO, 125 MHz).

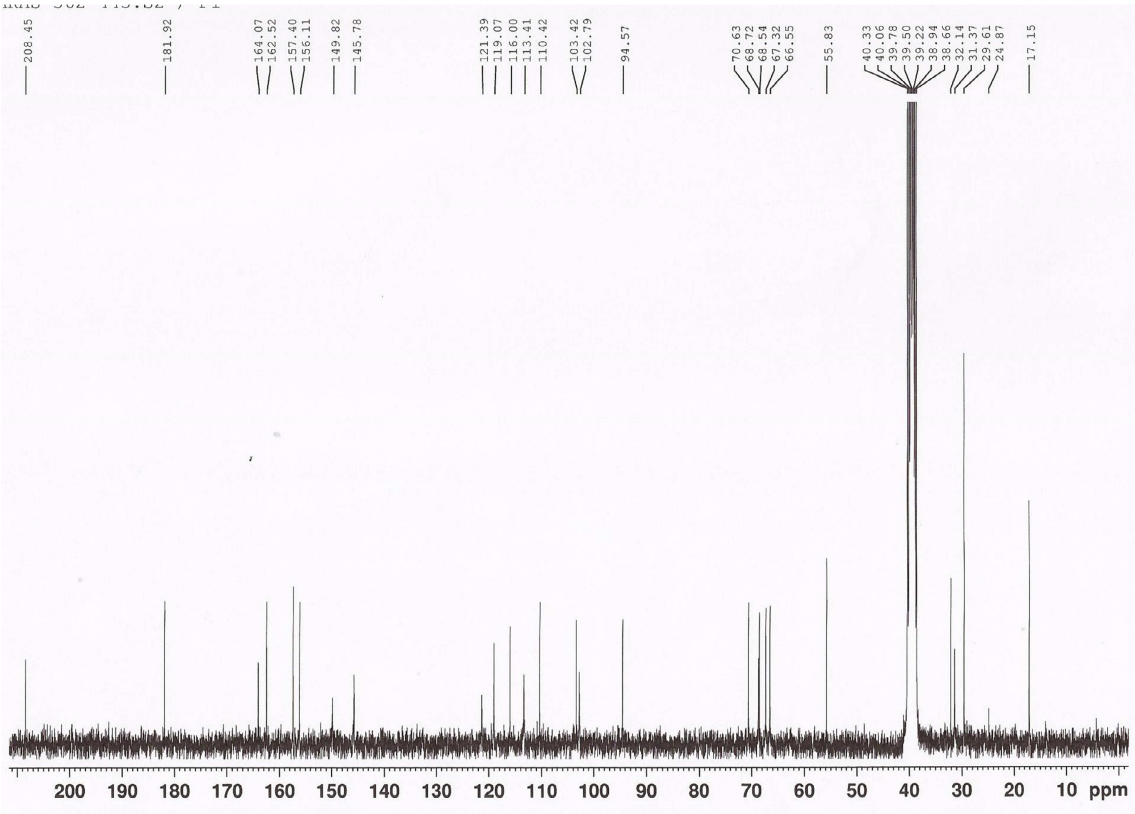

Figure S14. ^{1}H NMR spectrum of **2** (DMSO-*d6*, 500 MHz).

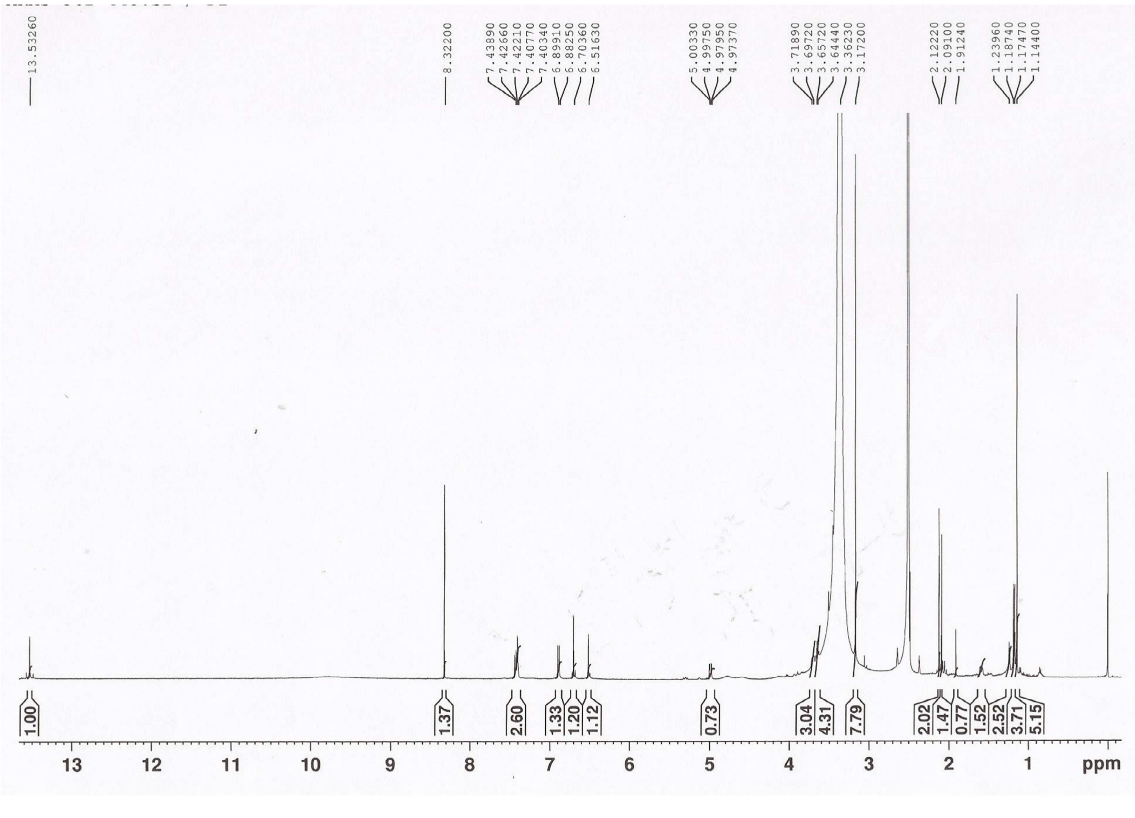

Figure S15. ^{13}C NMR spectrum of **2** (DMSO-*d6*,, 500 MHz).

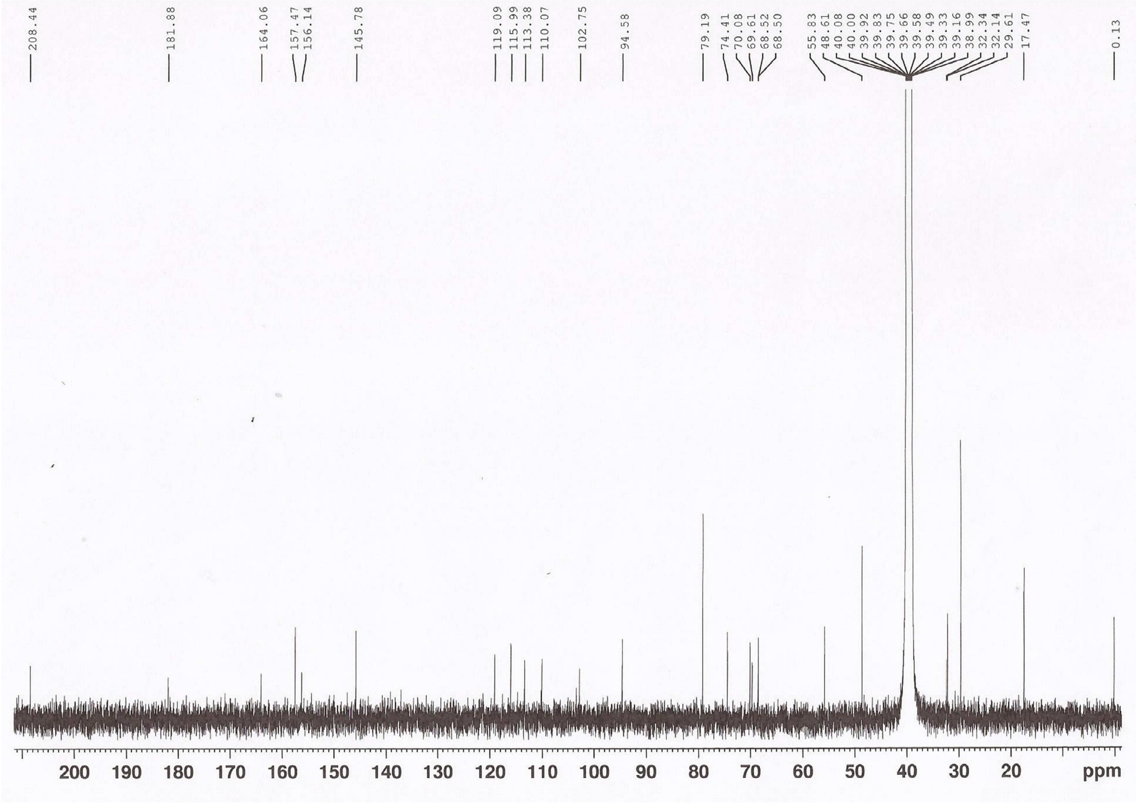

Figure S16. COSY spectrum of **2** (DMSO-*d6*, 500 MHz).

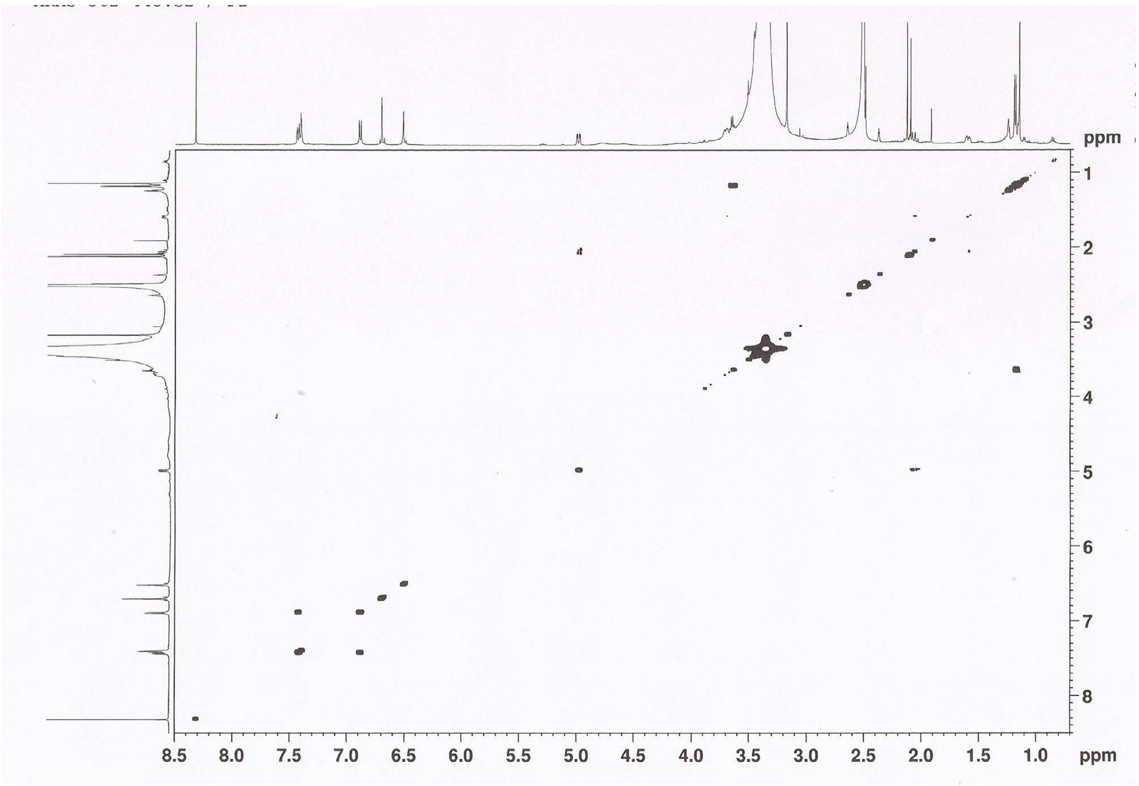

Figure S17. HSQC spectrum of **2** (DMSO-*d6*, 500 MHz).

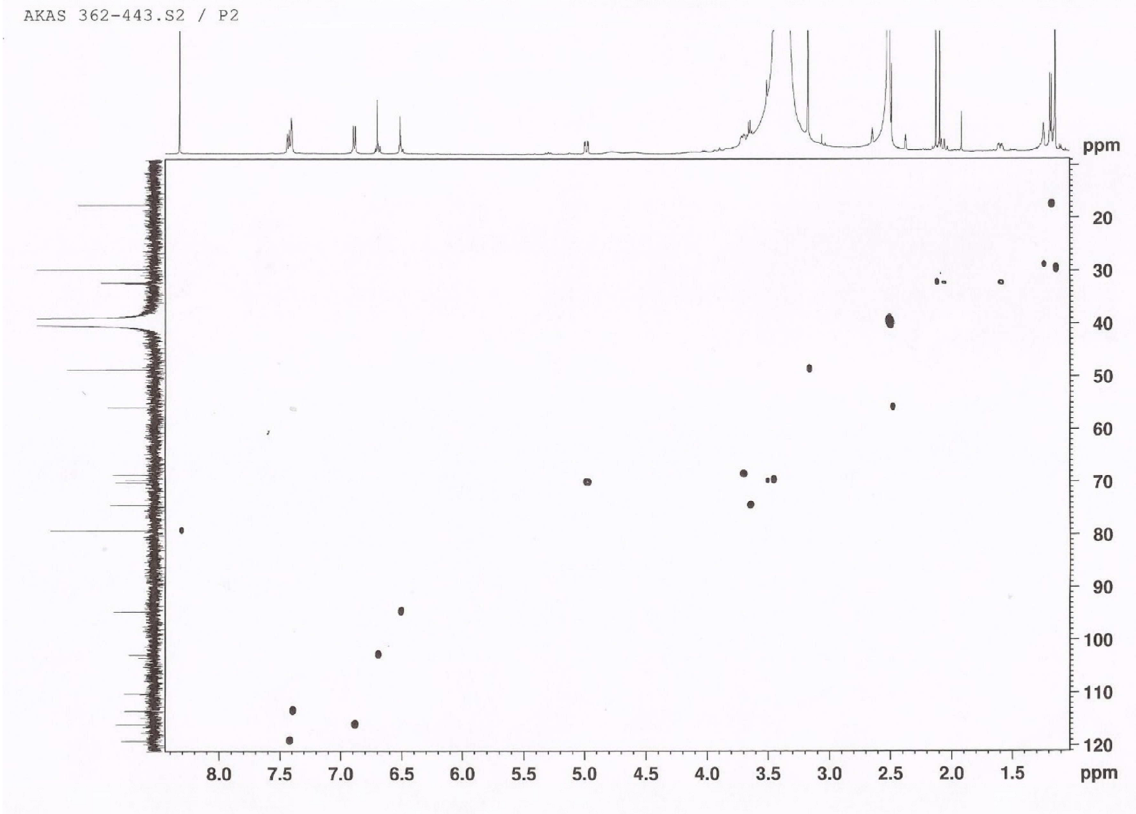

Figure S18. HMBC spectrum of **2** (DMSO-*d6*, 500 MHz).

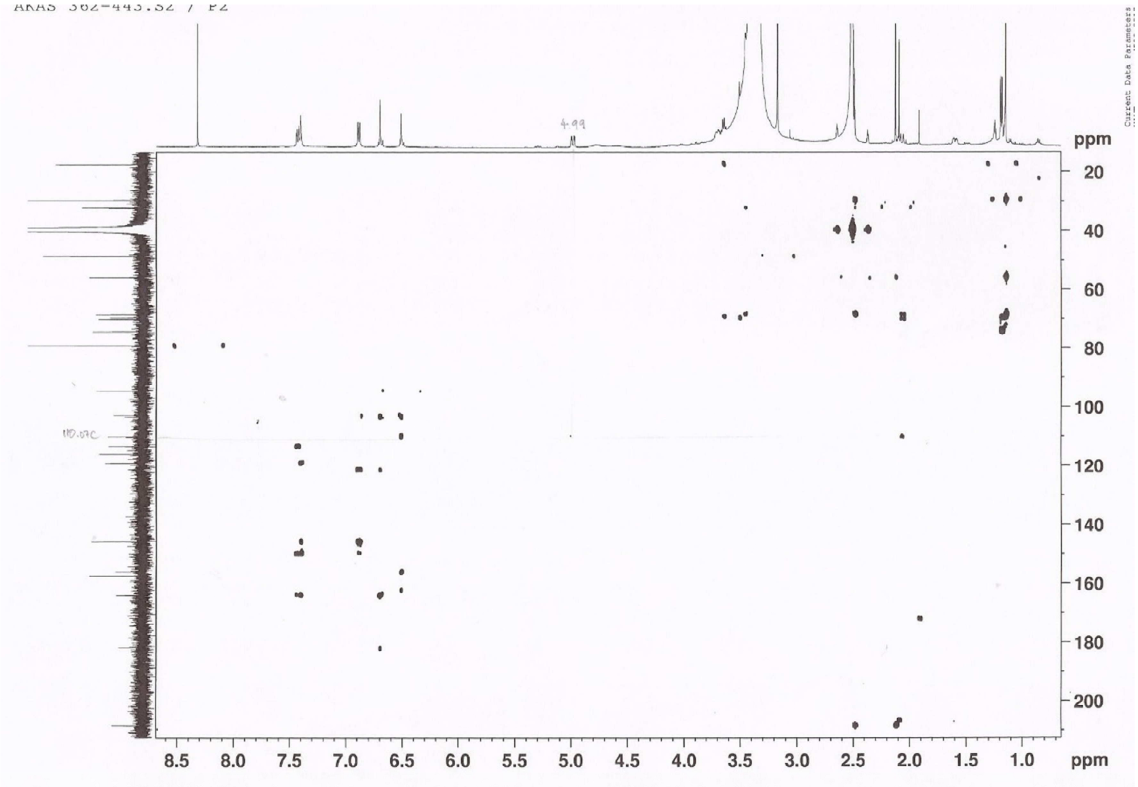

Figure S19. ^{1}H spectrum of **3a** (DMSO-*d6*, 500 MHz).

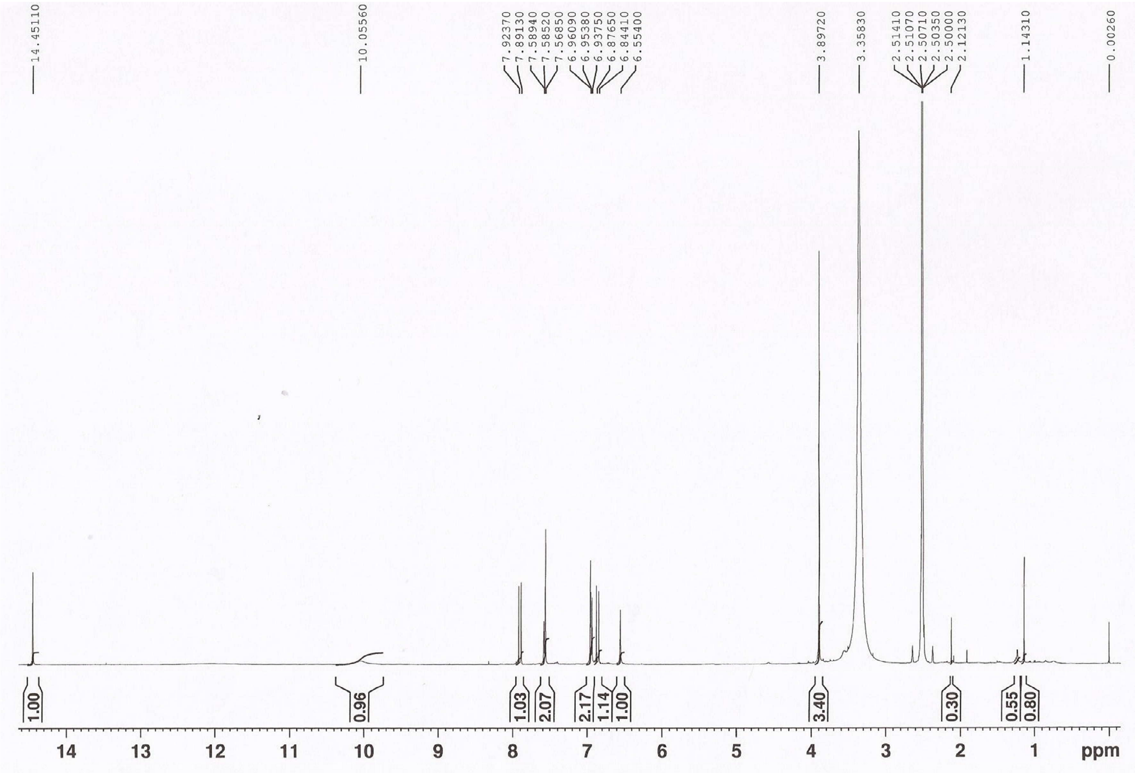

Figure S20. ^{13}C NMR spectrum of **3a** (DMSO-*d6*, 125 MHz).

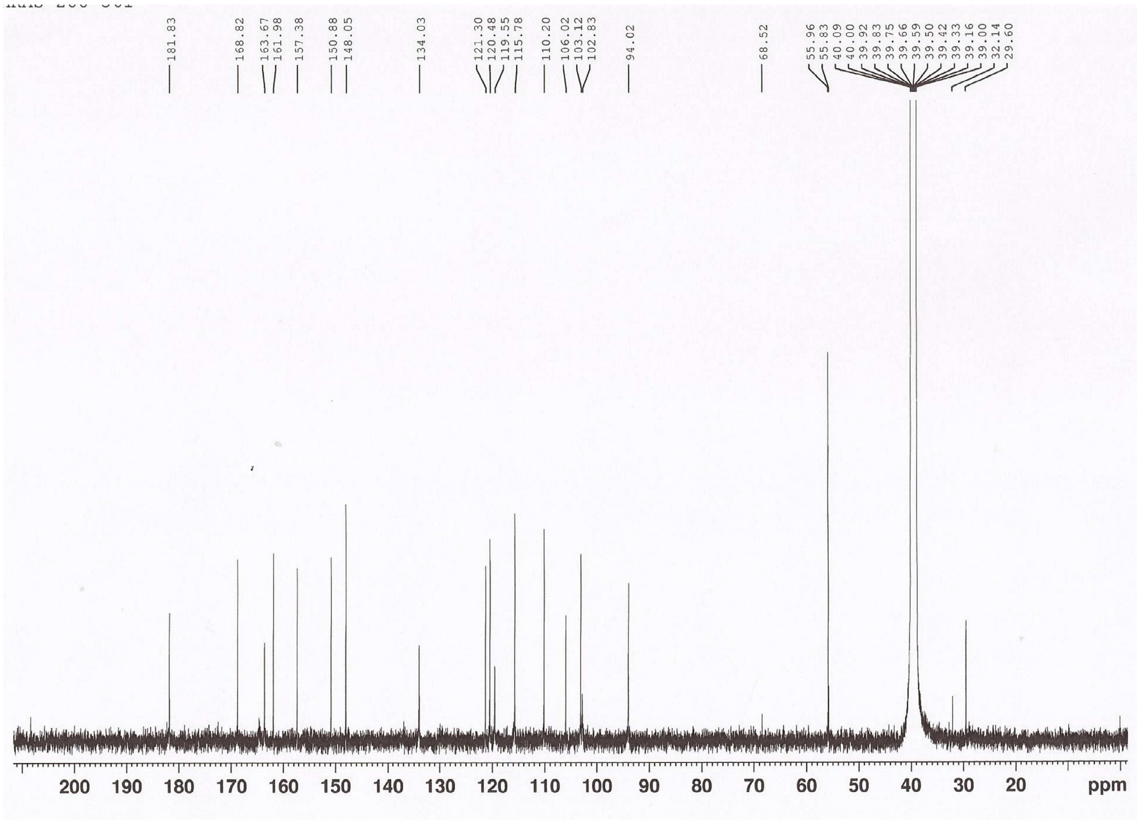

Figure S21. COSY spectrum of **3a** (DMSO-*d6*, 500 MHz).

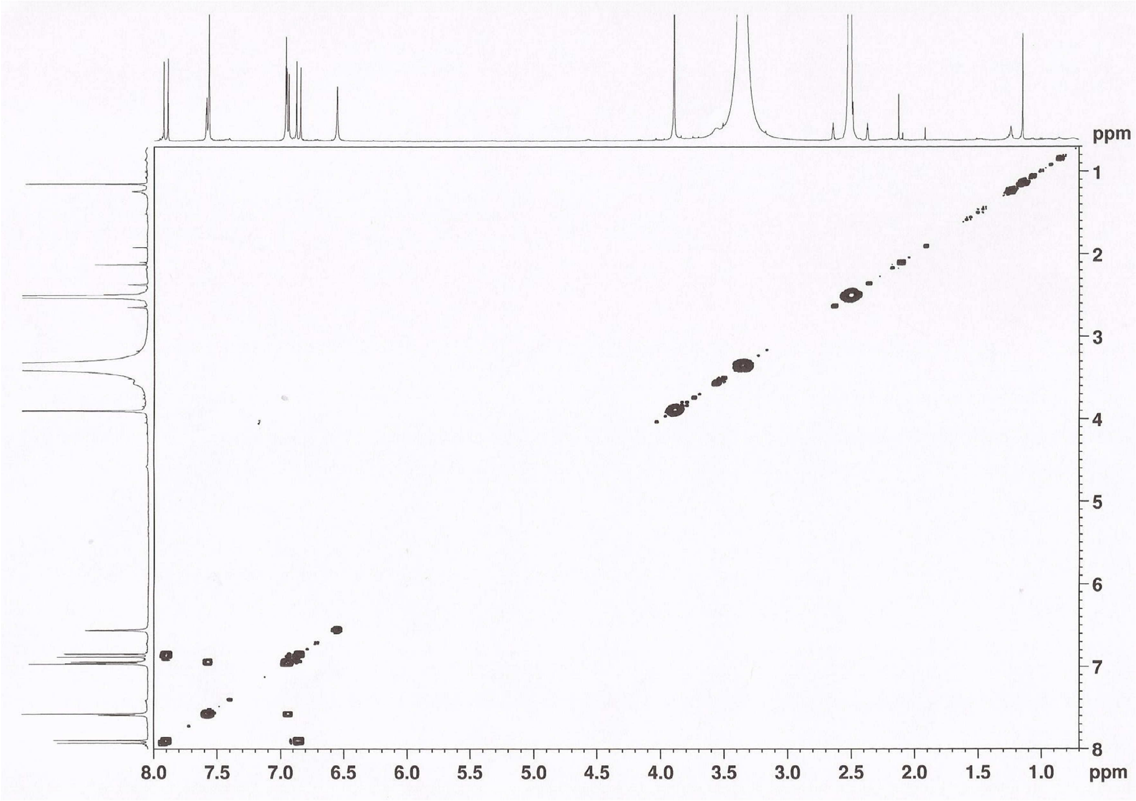

Figure S22. HSQC spectrum of **3a** (DMSO-*d6*, 500 MHz).

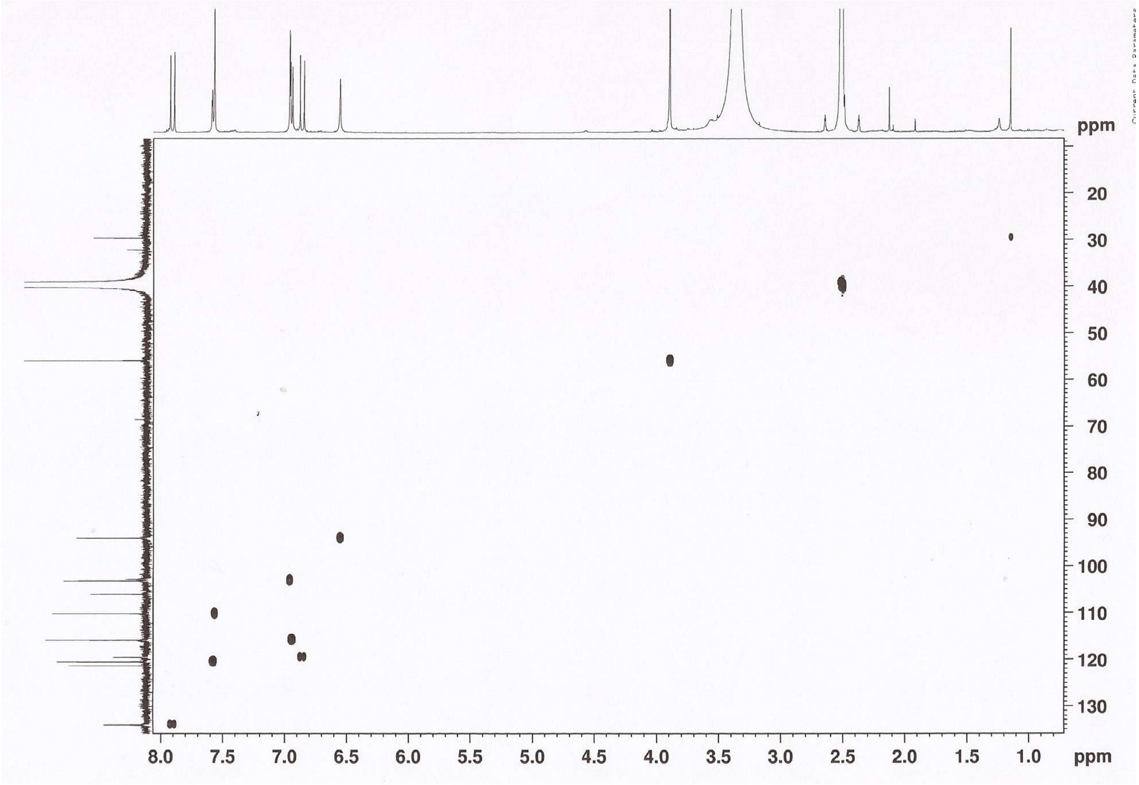

Figure S23. HMBC spectrum of **3a** (DMSO-*d6*, 500 MHz).

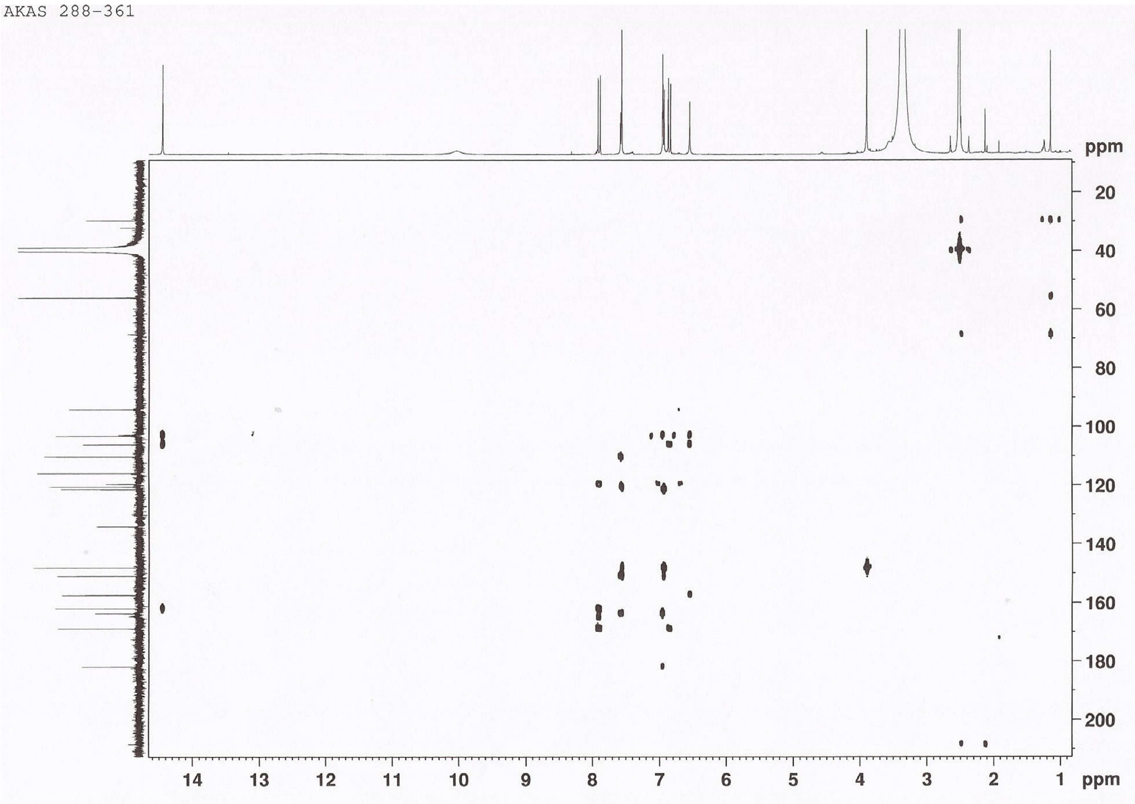

Figure S24. ^{1}H spectrum of **3b** (DMSO-*d6*, 500 MHz).

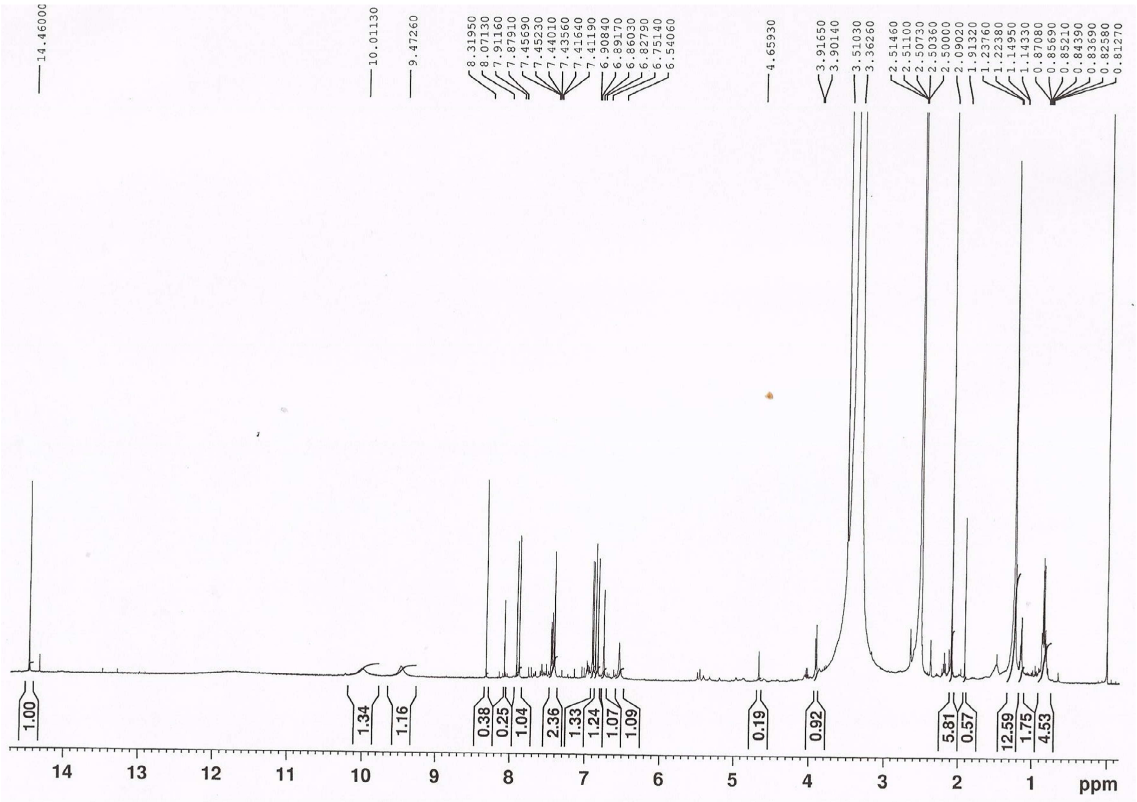

Figure S25. ^{13}C spectrum of **3b** (DMSO-*d6*, 500 MHz).

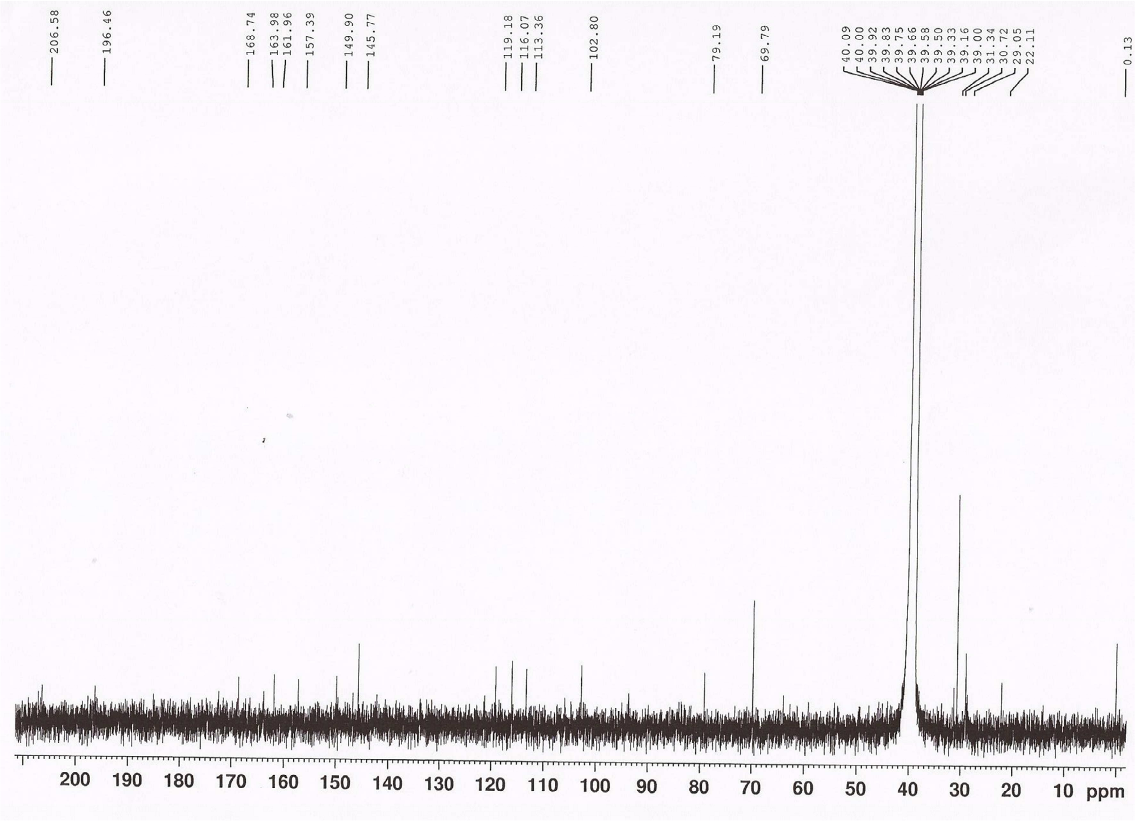

Figure S26. ^{1}H NMR spectrum of **4** (DMSO-*d6*, 500MHz).

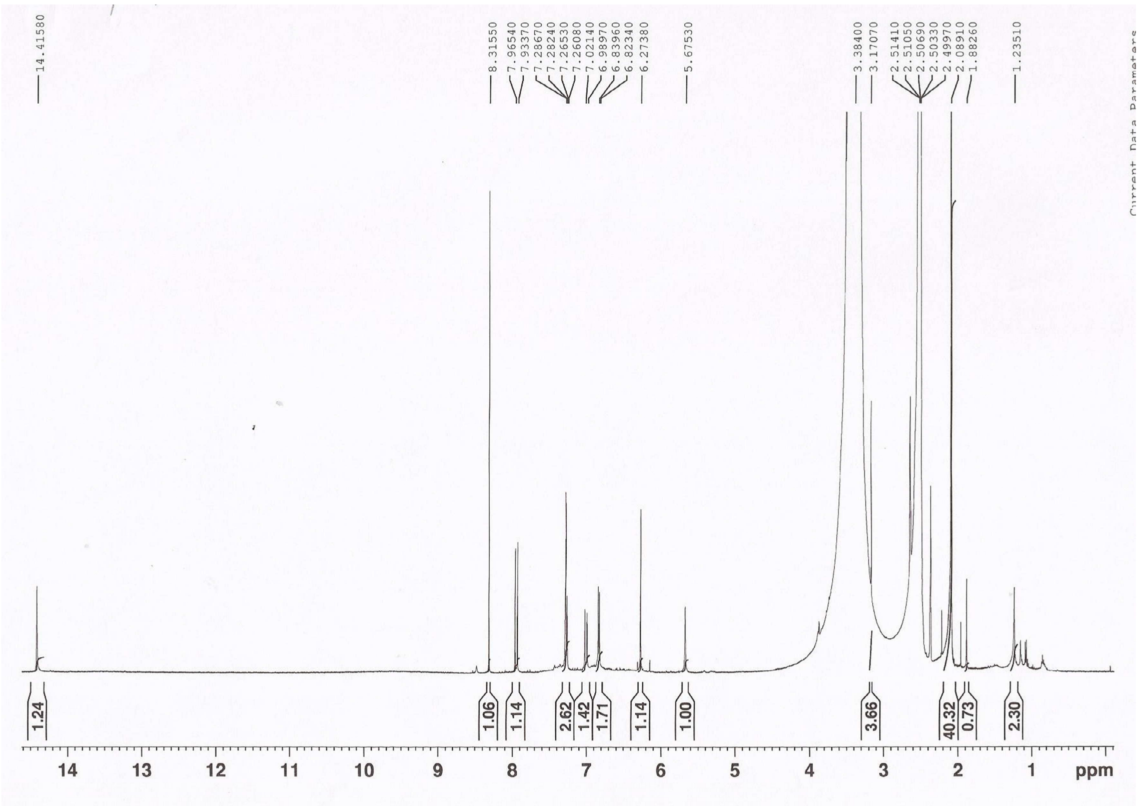

Figure S27. ^{13}C NMR spectrum of **4** (DMSO-*d6*, 125MHz).

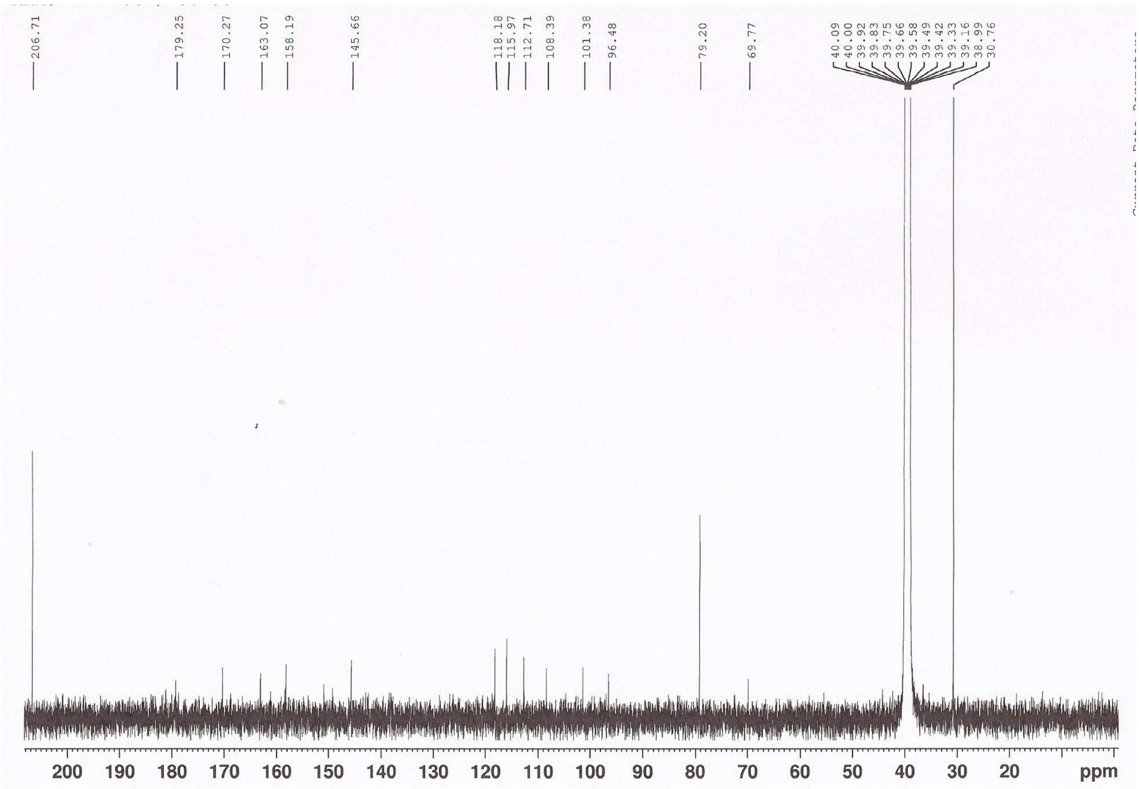

Figure S28. ^{1}H NMR spectrum of **5** (DMSO-*d6*, 300 MHz).

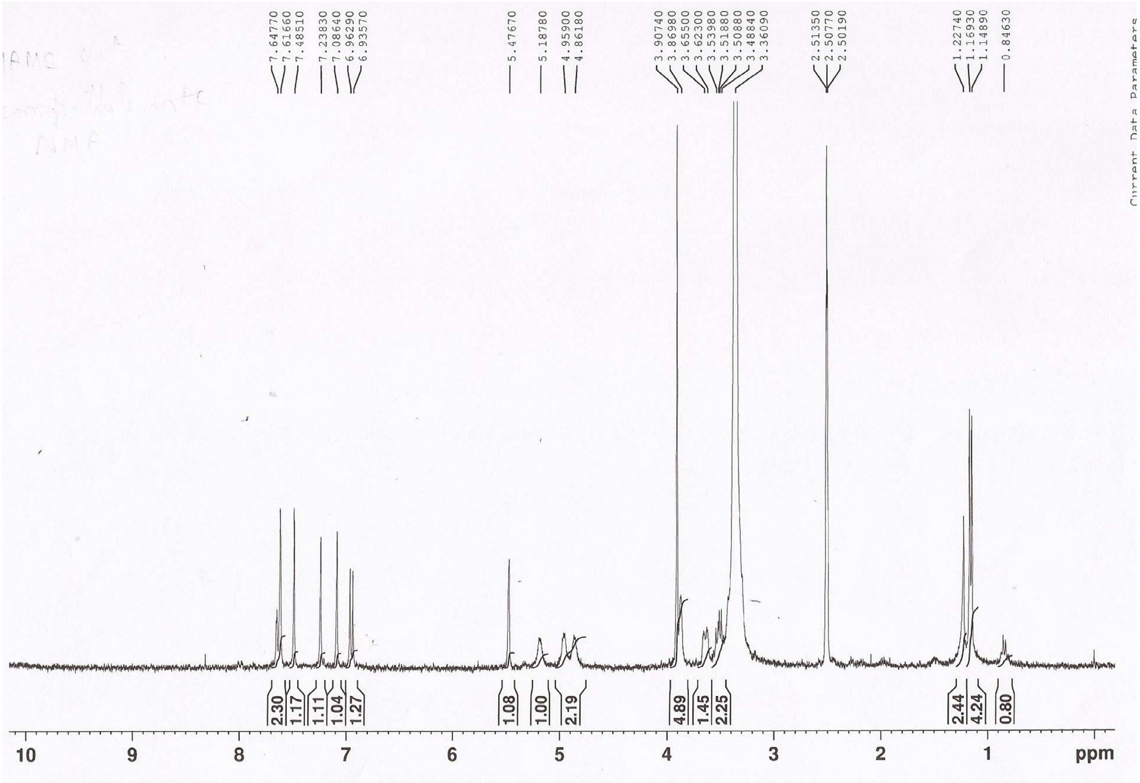

Figure S29. ^{13}C NMR spectrum of **5** (DMSO-*d6*, 75MHz).

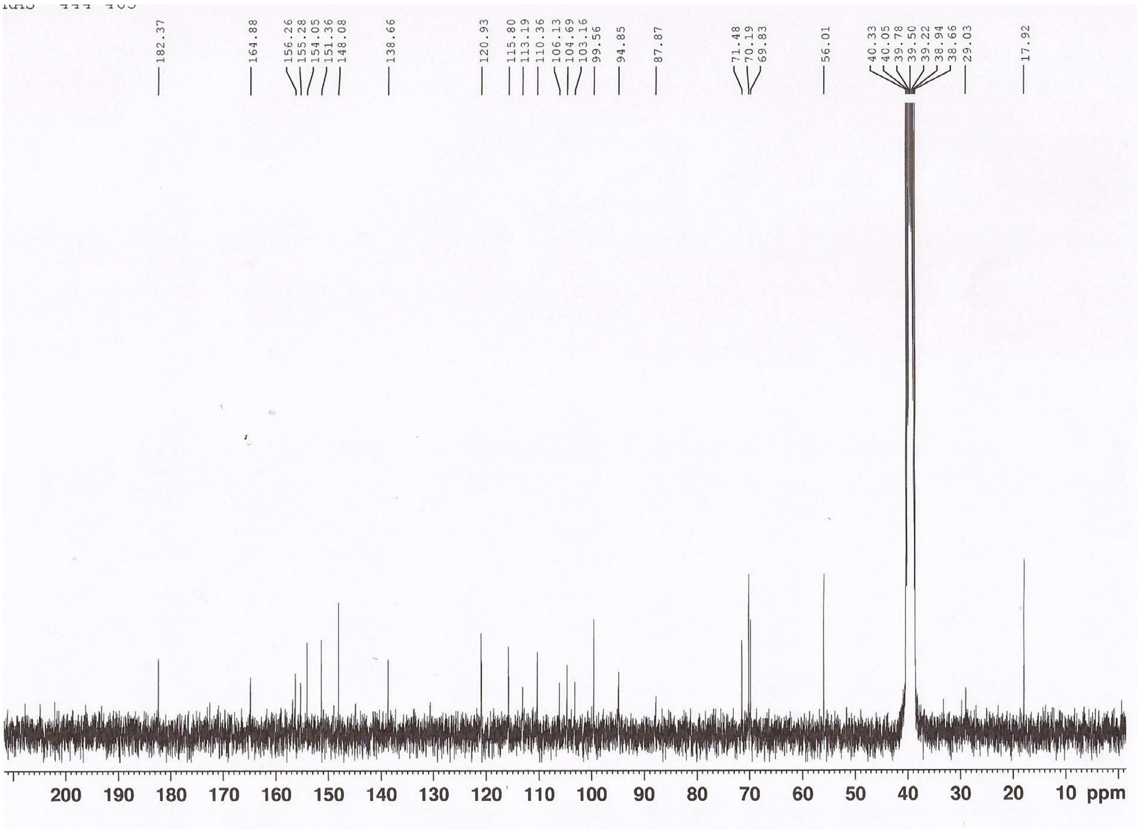

Figure S30. ORTEP view of **1a**.

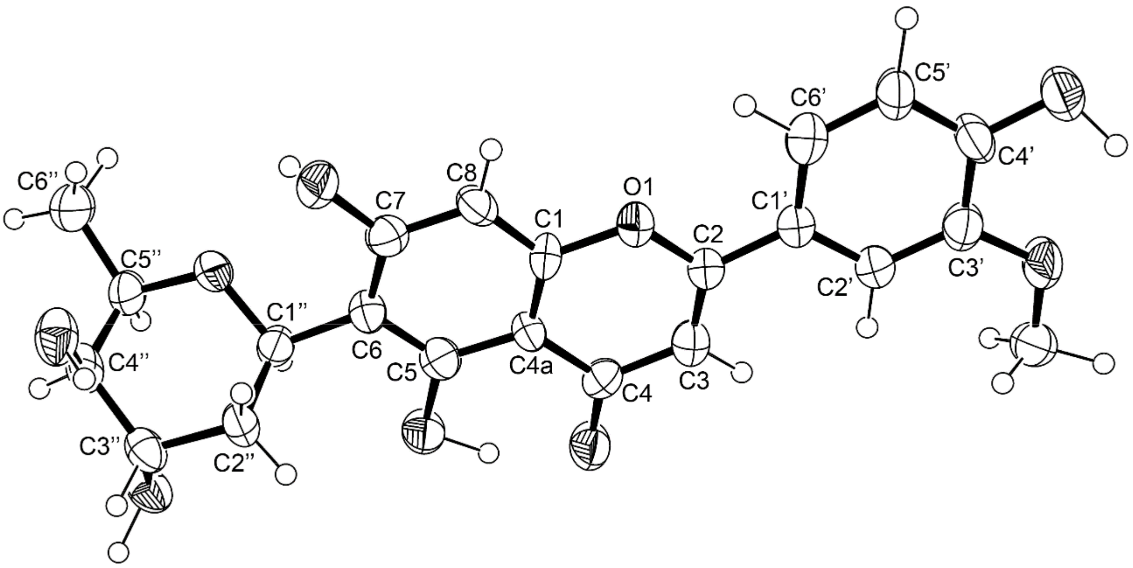

Supplement: Supplementary file 1 [file molecules-23-02202-s001.pdf]
